# Multimetallic nanoparticles decorated metal-organic framework for boosting peroxidase-like catalytic activity and its application in point-of-care testing

**DOI:** 10.1186/s12951-023-01946-8

**Published:** 2023-06-09

**Authors:** Pian Wu, Fangjie Gong, Xiangling Feng, Yong Xia, Lehuan Xia, Tianhan Kai, Ping Ding

**Affiliations:** 1 Xiang Ya School of Public Health, Central South University, Changsha, Hunan 410078 China; 2 Hunan Provincial Key Laboratory of Clinical Epidemiology, Changsha, Hunan 410078 China; 3 Affiliated Hospital of Xiangnan University, Chenzhou, Hunan 423000 China; 4 Chenzhou Third People’s Hospital, Chenzhou, Hunan 423000 China

**Keywords:** Nanozyme, Peroxidase-like, Multimetallic nanoparticles, Metal-organic frameworks, Point-of-care testing

## Abstract

Metal-organic frameworks (MOFs) are a sort of promising peroxidase-like nanozyme but face the challenge that the inorganic nodes in most of the MOF structures are generally blocked by the organic linkers. Further enhancement or activation of their peroxidase-like activity plays an important role in developing MOF-based nanozymes. Herein, a multimetallic nanoparticle (NP) decorated-MOF, Cu/Au/Pt NP decorated-Cu-TCPP(Fe) nanozyme (CuAuPt/Cu-TCPP(Fe)) was synthesized in situ and served as a peroxidase-like nanozyme. The peroxidase-like activity of this stable CuAuPt/Cu-TCPP(Fe) nanozyme was enhanced due to the decreased potential barriers for *OH generation in the catalytic process. Owing to the remarkable peroxidase-like activity, a CuAuPt/Cu-TCPP(Fe)-based colorimetric assay was established for the sensitive determination of H_2_O_2_ and glucose with the limit of detection (LOD) of 9.3 µM and 4.0 µM, respectively. In addition, a visual point-of-care testing (POCT) device was developed by integrating the CuAuPt/Cu-TCPP(Fe)-based test strips with a smartphone and was employed for a portable test of 20 clinical serum glucose samples. The results determined by this method agree well with the values deduced by clinical automatic biochemical analysis. This work not only represents an inspiration for the usage of MNP/MOF composite as a novel nanozyme for POCT diagnosis, but also provides a deeper insight and understanding into the enhanced enzyme-mimic effect of MNP-hybrid MOF composites, which in turn will guide the engineering of MOF-based functional nanomaterials.

**Graphical Abstract**

**Supplementary Information:**

The online version contains supplementary material available at 10.1186/s12951-023-01946-8.

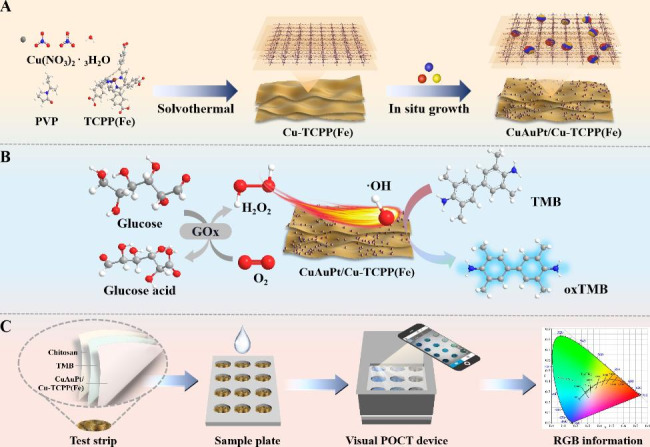

## Introduction

Nanozymes, as a promising alternative to natural enzymes, were defined as nanomaterials with intrinsic enzyme-mimic activity and have attracted extensive interest in the past decade as alternatives to natural enzymes [1, 2]. Combining the merits of conventional chemical catalysts and biocatalysts, nanozymes showed advantages in the tailored design, ease of fabrication, high yield, great stability, and low cost [3, 4]. According to the types of catalysis, nanozymes can be classified into peroxidase mimic, oxidase mimic, catalase mimic, and so on [5, 6]. Among them, the peroxidase mimics are the most widely designed and applied in biosensors, environmental protections, disease diagnosis, and clinic treatments [7]. A large number of nanomaterials with intrinsic peroxidase activity have been designed and developed, including but not limited to metals, metal oxides, and carbon-based materials [8, 9].

Metal-organic frameworks (MOFs), the promising nanozyme candidates, are sort of porous coordination polymers formed by the coordination of transition metal ions/clusters as nodes and organic ligands as linkers [10]. The regular arrangement of organic ligands and metal nodes in MOFs provides lots of accessible catalytic sites and endows the MOFs with intrinsic enzyme-mimicking properties [11]. Benefiting from their abundant active sites, tailorable properties, and favorable stability as well as excellent enzyme-like activity, a wide variety of research on the enzyme-mimicking characteristics, especially the peroxidase-mimicking activity, of the MOF-based nanomaterials are constantly emerging, such as MILs, PCNs, and ZIFs etc [12–15]. These MOFs with peroxidase-like activity could catalyze the decomposition of H_2_O_2_ into hydroxyl radical (•OH) and superoxide radical (•O_2_^−^), which shows great potential in development of catalytic-based biosensors, reactive oxygen species (ROS)-mediated bacteria, and tumor treatment [16, 17].

Despite the great development in peroxidase-like MOFs, these MOFs still face challenges that the inorganic nodes in most of the MOF structures are generally blocked by the organic linkers, which limits their full potential in peroxidase-mimic reactions [18]. To circumvent this limitation, one of the effective strategies is using the MOFs as the substrate to hybrid with the metal nanoparticles to endow the MOFs with synergistic catalytic effects to promote the peroxidase-like activity [19, 20]. The intrinsic enzyme-like activity nanoparticles, such as Au NPs, Pt NPs, and Cu NPs, have been integrated within MOFs and have demonstrated their enhanced peroxidase-like activity [21, 22]. For instance, Hu and co-workers in situ reduced the ultrasmall Au NPs on aluminum-based porphyrinic MOFs (UsAuNPs/MOFs) [23], Li and co-workers loaded Pt NPs on MIL-88B-NH_2_ (Pt/Fe-MOF) [24]. Both of UsAuNPs/MOFs and Pt/Fe-MOF showed the enhanced peroxidase-like activity, which confirmed that the hybridization of MOF with metal nanoparticles was an effective way to promote peroxidase-mimicking activity.

Considering that the multimetallic nanoparticles generally showed better enzyme-like activity than monometallic nanoparticles [25, 26], it is reasonable to predict that a better catalytic performance can be achieved using multimetallic NP-decorated MOFs. Several multimetallic-based nanozymes have been reported, such as Au@PtRu nanozyme [27], PtPdCu trimetallic nanoalloys [28], and NiCo_2_O_4_ mesoporous spheres [29], which exhibited much higher catalytic performance than that obtained from monometallic or bimetallic nanozymes alone. Our previous work demonstrate that Cu/Au/Pt trimetallic nanoparticles (Cu/Au/Pt TNPs) showed enhanced peroxidase-like enzyme activity compared to corresponding monometallic and bimetallic NPs [30–32]. Thus, we believe that growing Cu/Au/Pt TNPs onto MOFs could obtain a high-efficient peroxidase-like nanozyme.

Herein, a novel multimetallic NP-modified MOF nanozyme, CuAuPt/Cu-TCPP(Fe), was prepared via in situ synthesis of Cu/Au/Pt trimetallic nanoparticles on a 2D MOF (Cu-TCPP(Fe)) structure.

The enhanced peroxidase-like activity of the prepared complex was investigated via the calculation of the free energy of H_2_O_2_ decomposition steps using density functional theory (DFT). Based on the superior peroxidase-mimicking activity of CuAuPt/Cu-TCPP(Fe), the complex was utilized for the sensitive determination of H_2_O_2_ and glucose, which were two important substances involved in human physiological processes [33, 34]. The practical application of this CuAuPt/Cu-TCPP(Fe) nanozyme was demonstrated by developing a point-of-care testing (POCT) device using CuAuPt/Cu-TCPP(Fe)-based test strips with the smartphone for the portable detection of glucose in human serum samples. This work provides a deeper insight into the enhanced enzyme-mimic effect of metal NP-hybrid MOF composites, which thus in turn will guide the engineering of MOF-based functional nanomaterials. Besides that, the constructed POCT device based on metal NP-hybrid MOF composites shows great potential for personalized healthcare and clinical diagnosis.

## Results and discussion

### Synthesis and characterization of CuAuPt/Cu-TCPP(Fe)

**Fig. 1:**
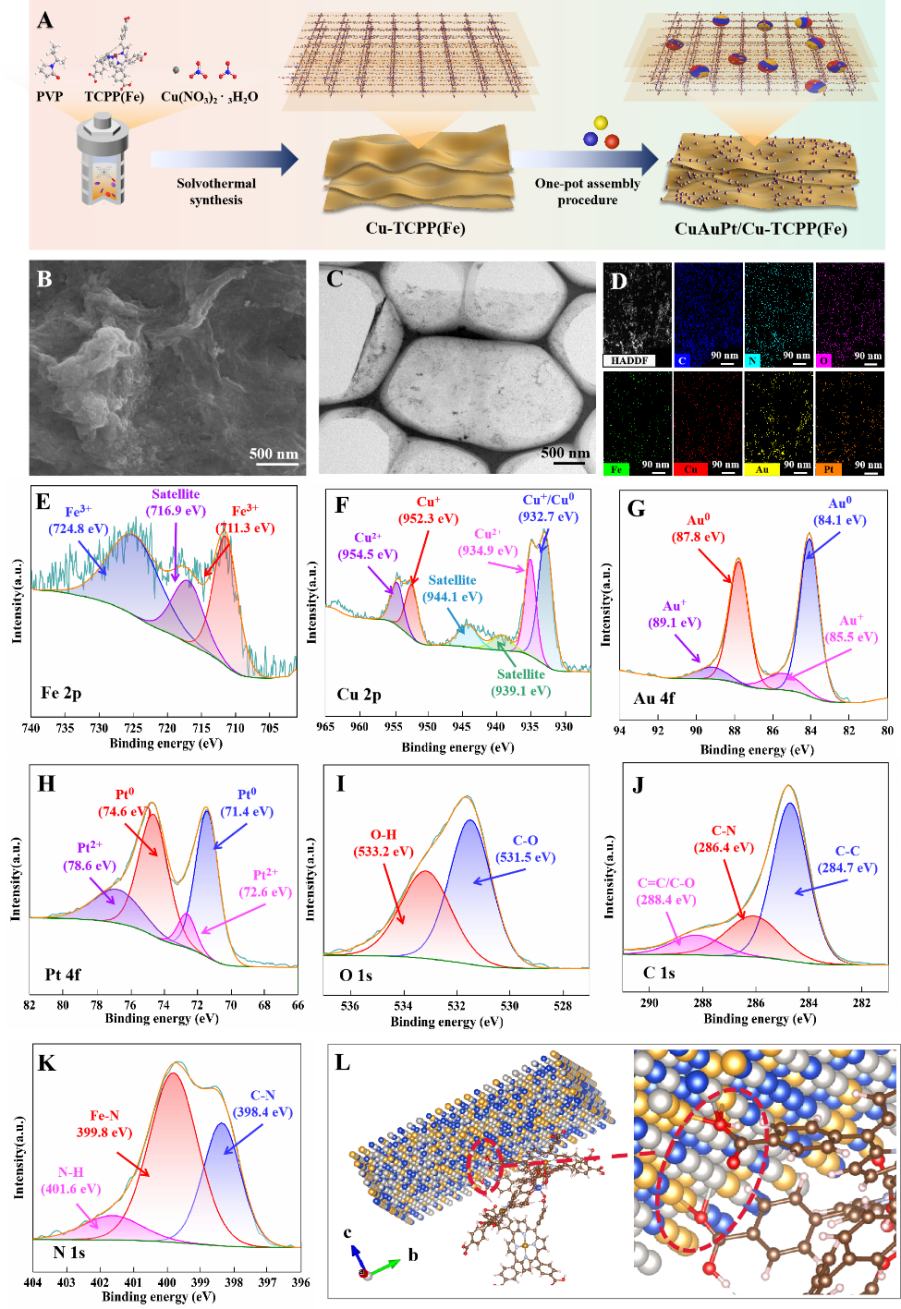
**(A)** Schematic illustration of the preparation CuAuPt/Cu-TCPP(Fe) nanozyme. **(B)** SEM, **(C)** TEM, and **(D)** Element mapping images of CuAuPt/Cu-TCPP(Fe) nanozyme. **(E-K)** High-resolution of Fe2p, Cu2p, Au4f, Pt4f, O1s, C1s, and N1s spectra of CuAuPt/Cu-TCPP(Fe) nanozyme, respectively. **(L)** Optimized structures of CuAuPt/Cu-TCPP(Fe) nanozyme

Considering that bulk MOFs usually suffer from the disadvantage of limited catalytic performance due to their slower diffusion rates of substrates [35]. Cu-TCPP(Fe), one of the ultrathin two-dimensional MOF nanosheets (2D-MOFs) with lower mass transfer resistance and more accessible active sites for catalytic reactions [36], was selected as an example to prepare Cu/Au/Pt TNP-modified MOFs. The Cu-TCPP(Fe) nanosheets were firstly synthesized using a solvothermal method. The SEM and TEM images showed that the resultant Cu-TCPP(Fe) nanosheets displayed a tulle-like structure with a smooth surface (Fig. S3). Then, as schematically presented in Fig. 1A, Cu-TCPP(Fe) nanosheets were applied as a supporting substance for the in situ growth of the Cu/Au/Pt TNPs to synthesize CuAuPt/Cu-TCPP(Fe) nanozyme by a one-pot assembly procedure.

In order to achieve better catalytic effect, we explored different feeding ratios of CuAuPt TNPs and Cu-TCPP(Fe). The results show that when the ratio of CuAuPt TNPs to Cu-TCPP(Fe) was equal to 3:1, the catalytic performance is the best (Fig. S4). Therefore, in the following experiments, we use this ratio to synthesize CuAuPt/Cu-TCPP(Fe). The SEM image revealed that the obtained CuAuPt/Cu-TCPP(Fe) retained a tulle-like structure but with a lot of small particles loading, resulting in a rough surface (Fig. 1B). TEM images of CuAuPt/Cu-TCPP(Fe) exhibited an ultrathin feature, and some darker spherical particles with the diameter of 3 ~ 5 nm were uniformly distributed on the sheets (Fig. 1C and Fig. S5A). The elemental mapping showed the C, N, O, Fe, Cu, Au, and Pt elements uniformly existed in CuAuPt/Cu-TCPP(Fe) complex (Fig. 1D). The high-resolution TEM (HRTEM) images exhibited that the well-defined lattice fringes of these dark spherical particles ranged from 0.208 to 0.247 nm, which were coincidentally located between those of Cu (111) (0.208 nm, JCPDS-04-0836), Au (111) (0.235 nm, JCPDS-04-0784), and Pt (111) (0.226 nm, JCPDS-04-0802) crystal facets, respectively, indicating that dark spherical particles were alloyed Cu/Au/Pt TNPs instead of monometallic nanoparticles (Fig. S5B). The crystal structure of CuAuPt/Cu-TCPP(Fe) was further investigated by XRD analysis (Fig. S6). Compared to the low crystallinity of single Cu-TCPP(Fe) [37], the CuAuPt/Cu-TCPP(Fe) showed four diffraction peaks at 39.0°, 44.9°, and 65.6° which were indexed to the (111), (200), (220) facets, respectively, and one broad peak at 20.0° which corresponded to amorphous were observed, demonstrating that Cu/Au/Pt TNPs was successfully grown on Cu-TCPP(Fe) nanosheets [38, 39]. In addition, the TG analysis showed the whole weight loss of CuAuPt/Cu-TCPP(Fe) was about 28.31% while that of Cu-TCPP(Fe) was 83.72%, which was attributed to the introduction of Cu/Au/Pt TNPs (Fig. S7). The ICP-MS result exhibited that the atomic ratio of Fe: Cu: Au: Pt was 2.64: 12.50: 38.15: 46.71 (Table S1). These results demonstrated that the CuAuPt/Cu-TCPP(Fe) nanozyme was successfully prepared.

To analyze the chemical composition and structure of CuAuPt/Cu-TCPP(Fe), the XPS was performed (Fig. S8 and Fig. 1E-K). The XPS survey spectrum showed that the C, N, O, Fe, Cu, Au, and Pt elements existed in CuAuPt/Cu-TCPP(Fe), and the peaks corresponded to C1s, N1s, O1s, Fe2, Cu2p, Au4f, and Pt4f, respectively (Fig. S8). The high-resolution spectra of Fe2p showed the characteristic peaks at 711.3 eV (Fe2p_3/2_) and 724.8 eV (Fe2p_1/2_), suggesting Fe^3+^ existed in CuAuPt/Cu-TCPP(Fe) and the introduction of Cu/Au/Pt TNPs did not change the phase state of Fe (Fig. 1E) [40]. In the high-resolution XPS of Cu2p, the peak at 932.7 eV (Cu2p_3/2_) belonged to Cu^0^/Cu^+^, and the peak at 952.3 eV (Cu2p_1/2_) was ascribed to Cu^+^, while the peak pair at 934.9 and 954.5 eV corresponded to Cu^2+^ species [41, 42] (Fig. 1F). The high-resolution XPS of Au4f showed a strong peak pair at 84.1 eV (4f7/2) and 87.8 eV (4f5/2) arose from Au^0^, and another peak pair at 85.5 eV and 89.1 eV attributed to Au^+^, respectively (Fig. 1G) [43, 44]. For the high-resolution XPS of Pt4f, the peak pair at 71.4 eV (4f7/2) and 74.6 eV (4f5/2) were assigned to Pt^0^, while the peak pair at 72.6 eV and 76.8 eV were originated from Pt^2+^ (Fig. 1H) [45, 46]. Besides, the XPS data in Table S2 showed that metallic Cu^0^, Au^0^, and Pt^0^ are the main components in Cu/Au/Pt alloys and the introduction of Cu/Au/Pt alloys did not change the Fe phase state of Cu-TCPP(Fe).

To further reveal the interaction between Cu/Au/Pt TNPs and Cu-TCPP(Fe), the high-resolution XPS spectra, O1s, C1s, and N1s, of CuAuPt/Cu-TCPP(Fe) were analyzed. Compared to the Cu-TCPP(Fe) nanosheets [37, 40], in the high-resolution XPS spectra of O1s, the peaks ascribed to C = O and O-H shifted towards the lower binding energy to 531.5 eV and 533.2 eV in CuAuPt/Cu-TCPP(Fe), respectively (Fig. 1I). Moreover, the FTIR spectra showed that the peak correspond to C = O stretching vibration in -COOH groups was shifted from at 1684 cm^− 1^ to 1660 cm^− 1^ after in situ growth of Cu/Au/Pt TNPs on Cu-TCPP(Fe) (Fig. S9). The binding energy changed in C = O and O-H, and the infrared absorption peak shifted in C = O suggesting that -COOH was the coordinate bond to connect the Cu/Au/Pt TNPs and Cu-TCPP(Fe). For the high-resolution XPS of C1s, the peak attributed to the C-N bond shifted to lower binding energy (286.1 eV), and a new peak attributed to π-π satellite (290.1 eV) emerged (Fig. 1J). Similarly, the peak at 399.5 eV (Fe-N) in the high-resolution XPS of N1s shifted to 399.8 eV and a new peak at 401.6 eV corresponding to N-H was observed in the high-resolution XPS spectra of N1s (Fig. 1K) [42, 47]. Herein, based on the analysis of XPS and FTIR, the optimized structural configurations of Cu/Au/Pt TNPs, Cu-TCPP(Fe), and CuAuPt/Cu-TCPP(Fe) nanozyme was presented in Fig. S10, Fig. S11, and Fig. 1L, respectively.

### Evaluation of the peroxidase-like activity of CuAuPt/Cu-TCPP(Fe) nanozyme

**Fig. 2:**
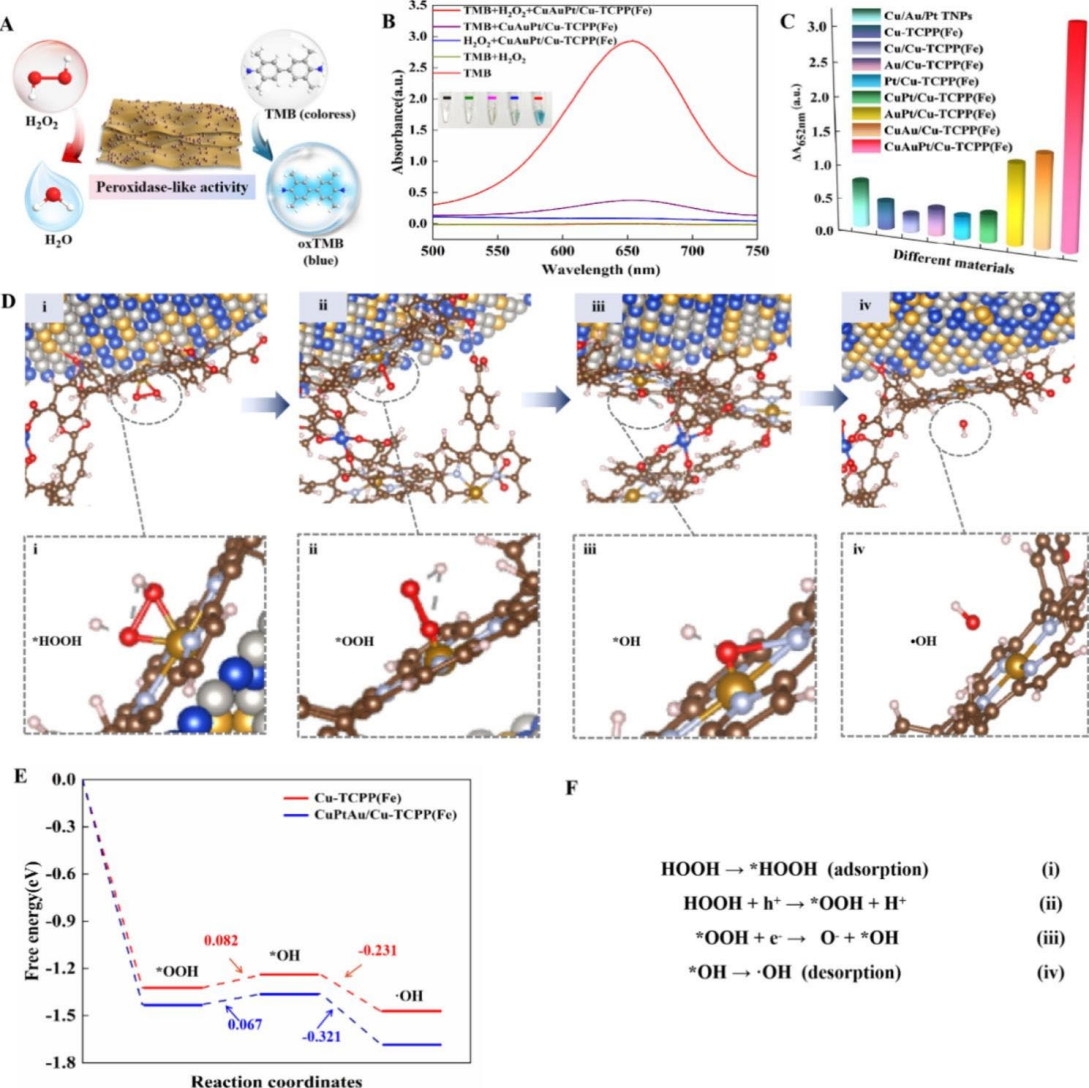
Evaluation and mechanism of the peroxidase-like activity of CuAuPt/Cu-TCPP(Fe) nanozyme. **(A)** Schematic presentation of peroxidase-like activity for CuAuPt/Cu-TCPP(Fe)nanozyme. **(B)** UV-vis absorption spectra of TMB with the addition of different samples, inset: chromogenic reaction of TMB. **(C)** Comparison of peroxidase-like activities among different materials. **(D)** DFT calculation for the catalytic steps of H_2_O_2_ by CuAuPt/Cu-TCPP(Fe) nanozyme. **(E)** Corresponding free energy diagram for peroxidase-like reaction on CuAuPt/Cu-TCPP(Fe) nanozyme. **(F)** The reaction mechanisms

As a peroxidase-like nanozyme, the peroxidase-like catalytic properties of CuAuPt/Cu-TCPP(Fe) nanozyme were investigated with the oxidation of TMB in the presence of H_2_O_2_ (Fig. 2A). As exhibited in Fig. 2B, the CuAuPt/Cu-TCPP(Fe) nanozyme could readily oxidize the colorless TMB into blue oxidized TMB (oxTMB) in the presence of H_2_O_2_, and the characteristic absorption peak appeared at 652 nm. However, when in the absence of CuAuPt/Cu-TCPP(Fe) nanozyme or H_2_O_2_, there was an invisible color change and a weak oxTMB absorption peak appeared. The results proved that the CuAuPt/Cu-TCPP(Fe) had a peroxidase-like activity.

To study the enhanced peroxidase-like activity of CuAuPt/Cu-TCPP(Fe) nanozyme, after optimization of the reaction conditions (Fig. S12), a catalytic comparison study was carried out among Cu/Au/Pt TNPs, Cu-TCPP(Fe), monometallic nanoparticles-modified Cu-TCPP(Fe), bimetallic nanoparticle-modified Cu-TCPP(Fe), and CuAuPt/Cu-TCPP(Fe). As shown in Fig. 2C and Fig. S13, all materials displayed a peroxidase-like activity, but the CuAuPt/Cu-TCPP(Fe) nanozyme presented the best catalytic performance among all materials, which confirmed that the introduction of Cu/Au/Pt TNPs onto Cu-TCPP(Fe) significantly promoted the peroxidase-like activity. For quantitative comparison, the absorbance difference value at 652 nm (ΔA_652nm_) in CuAuPt/Cu-TCPP(Fe) system was as a reference (100%), thus ΔA_652nm_ in the Cu/Au/Pt TNPs, Cu-TCPP(Fe), Cu/Cu-TCPP(Fe), Au/Cu-TCPP(Fe), Pt/Cu-TCPP(Fe), CuAu/Cu-TCPP(Fe), CuPt/Cu-TCPP(Fe), and AuPt/Cu-TCPP(Fe) was calculated to be 33.3%, 56.4%, 9.9%, 19.6%, 16.4%, 66.5%, 20.3%, and 58.1%, respectively. The catalytic ability of CuAuPt/Cu-TCPP(Fe) was improved by at least 30% under the same experimental conditions.

The catalytic performance of CuAuPt/Cu-TCPP(Fe) nanozyme was further studied by the enzyme kinetics theory. The Michaelis-Menten curve of CuAuPt/Cu-TCPP(Fe) nanozyme was obtained by monitoring the absorbance changes of products in a specified concentration range of TMB or H_2_O_2_. As can be seen from Fig. S14, the rate of catalytic reaction increased with the concentration increasing. The Michaelis constants (K_m_) and maximum reaction rates (V_max_) were calculated according to the Lineweaver-Burk plots (Table S3), and the results showed that the K_m_ of CuAuPt/Cu-TCPP(Fe) nanozyme for both TMB and H_2_O_2_ were much smaller than other reported peroxidase-like nanozymes, demonstrating that CuAuPt/Cu-TCPP(Fe) nanozyme had a better affinity for TMB and H_2_O_2_, and providing strong evidence that the CuAuPt/Cu-TCPP(Fe) nanozyme was greatly in favor of the activation of H_2_O_2_, and thus achieve the excellent peroxidase-like activity.

### Mechanism of CuAuPt/Cu-TCPP(Fe) as boosting peroxidase-like nanozyme

To better understand the boosting peroxidase-like activity of CuAuPt/Cu-TCPP(Fe) nanozyme, the catalytic mechanism studies was conducted. It has been reported that peroxidase mimetics always originate from the catalytic decomposition of H_2_O_2_ into •OH [48]. Therefore, electron paramagnetic resonance (EPR) spectroscopy was performed to verify the production of •OH [49]. As shown in Fig. S15A, there was no noticeable EPR signal in CuAuPt/Cu-TCPP(Fe) or H_2_O_2_ alone, but a characteristic 1:2:2:1 signal appeared in CuAuPt/Cu-TCPP(Fe) + H_2_O_2_, which corresponded to the 5,5-dimethyl-1-pyrroline N-oxide (DMPO) captured •OH, indicating that the CuAuPt/Cu-TCPP(Fe) could catalyze H_2_O_2_ to generate •OH. Meanwhile, the fluorescent experiments were conducted to determine the formation of •OH, in which the terephthalic acid (TA) was used as a fluorescent probe because 2-hydroxy terephthalic acid (TA-OH), the reaction product between TA and •OH, has a characteristic fluorescence emission peak at about 435 nm [50]. From Fig. S15B, the fluorescence intensity of CuAuPt/Cu-TCPP(Fe) + TA + H_2_O_2_ was significantly enhanced at 435 nm, while a negligible fluorescence was observed in TA, TA + H_2_O_2_, CuAuPt/Cu-TCPP(Fe), and CuAuPt/Cu-TCPP(Fe) + TA, indicating that the •OH was generated during the reaction. However, when we use tert-Butanol (TBA) to consume the produced •OH [51], the peaks of blue-colored oxTMB at 652 nm disappeared in TBA + CuAuPt/Cu-TCPP(Fe) + TMB + H_2_O_2_ group, suggesting that the catalytic ability of CuAuPt/Cu-TCPP(Fe) was inhibited by the addition of TBA, which demonstrated that •OH was the only free radical involved in the oxidation of TMB to oxTMB by CuAuPt/Cu-TCPP(Fe)-H_2_O_2_ catalytical system (Fig. S15C).

To systemically investigated the mechanism of the enhanced peroxidase-like activity of CuAuPt/Cu-TCPP(Fe) nanozyme, DFT calculation was carried out to understand the catalytic activation mechanism of H_2_O_2_ by CuAuPt/Cu-TCPP(Fe) nanozyme. As presented in Fig. 2D-E, at the beginning, the H_2_O_2_ molecule was absorbed on the center of iron (III) porphyrin (i), and the O-H bond was heterogeneously cleaved to form *OOH (ii). Next, the *OOH was cleaved to form *OH species (iii), followed by the desorption of *OH from the center of iron (III) porphyrin (iv). In step (iii), the energy barrier for the *OOH generates electrons *OH of CuAuPt/Cu-TCPP(Fe) was lower than that for Cu-TCPP(Fe) (0.067 versus 0.082 eV), indicating to the production of *OH is favorable when CuAuPt/Cu-TCPP(Fe) nanozyme was utilized. Finally, the •OH is generated from *OH. The decomposition process of H_2_O_2_ was presented in Fig. 2F. The DFT results illustrated that the CuAuPt/Cu-TCPP(Fe) nanozyme could decrease the potential barriers of intermediates, which resulted in a high-efficiency peroxidase-like catalytic activity.

### Detection of H_2_O_2_ and glucose by CuAuPt/Cu-TCPP(Fe)-based colorimetric assay

**Fig. 3:**
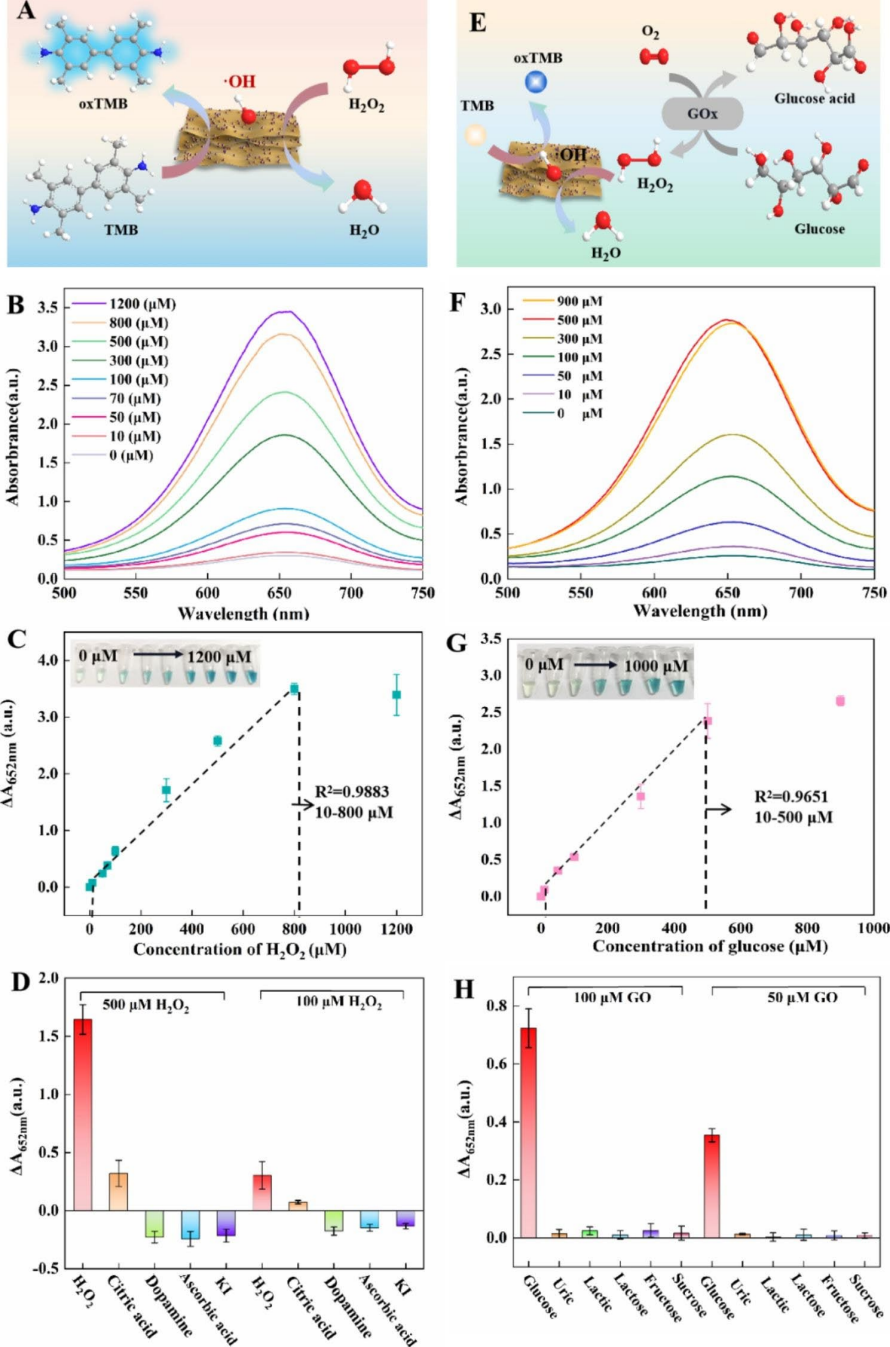
**(A)** Schematic illustration of the CuAuPt/Cu-TCPP(Fe)-based colorimetric assay for the detection of H_2_O_2_. **(B)** The absorption spectra of different H_2_O_2_ concentrations in the range from 0-1200 µM). **(C)** The linear curve of Δ_652nm_ for different H_2_O_2_ concentrations, inset: photos of TMB in presence of different H_2_O_2_ concentrations. **(D)** Specificity analysis of CuAuPt/Cu-TCPP(Fe)-based colorimetric assay for the H_2_O_2_ detection. **(E)** Schematic illustration of the CuAuPt/Cu-TCPP(Fe)-based colorimetric assay for the detection of glucose. **(F)** The absorption spectra of different glucose concentrations in the range from 0-900 µM), inset: photos of TMB in presence of different glucose concentrations. **(G)** The linear curve of Δ_652nm_ for different glucose concentrations, inset: photos of TMB in presence of different glucose concentrations. **(H)** Specificity analysis of CuAuPt/Cu-TCPP(Fe)-based colorimetric assay for glucose detection

Benefit from the excellent catalytic performance of the CuAuPt/Cu-TCPP(Fe) nanozyme, the CuAuPt/Cu-TCPP(Fe) nanozyme was applied for colorimetric detection of H_2_O_2_ (Fig. 3A). As shown in Fig. 3B, the A_652nm_ was increased gradually with the increasing concentrations of H_2_O_2_. A calibration linearity curve in the buffer was established as ΔA_652nm_ = 0.00454*C*_*H2O2*_ + 0.06478 (ΔA = A_H2O2_-A_blank_) with the H_2_O_2_ concentration ranging from 10 to 800 µM (R^2^ = 0.9883) (Fig. 3C). The limit of detection (LOD) was calculated to be 9.3 µM (3s/k). We compared multiple detection platforms, including different nanozymes and detection methods. Notably, this CuAuPt/Cu-TCPP(Fe)-based colorimetric detection of H_2_O_2_ displayed a wider linear range or lower LOD than other reported nanozyme-based colorimetric methods. At the same time, the CuAuPt/Cu-TCPP(Fe) colorimetry was lower than that of other nanozymes. The linear range of CuAuPt/Cu-TCPP(Fe) colorimetric method is better than electrochemical detection (Table S5). The selectivity of H_2_O_2_ was studied by using citric acid, dopamine, NO_3_^−^, ascorbic acid, and KI as interferences. It can be seen from Fig. 3D and Fig. S16 that H_2_O_2_ showed the highest absorbance intensity in both concentrations of 100 µM and 500 µM, which proved the CuAuPt/Cu-TCPP(Fe)-based colorimetric assay had a good selectivity in the detection of H_2_O_2_.

The glucose was also determined by the CuAuPt/Cu-TCPP(Fe)-based colorimetric assay by the introduction of glucose oxidase (GOx). As shown in Fig. 3E, the glucose was firstly incubated with GOx to generate gluconic acid and H_2_O_2_, then the generated H_2_O_2_ was detected by CuAuPt/Cu-TCPP(Fe)-based colorimetric system. Under the optimal incubation conditions with the GOx concentration of 8 mg/mL and the incubation time of 15 min (Fig. S17), we can found A_652nm_ increased gradually with glucose concentration increasing (Fig. 3F). The △A_652nm_ was linearly related to *C*_glucose_ in the range of 10 µM to 500 µM and the linear equation was △A_652nm_ = 0.00489*C*_glucose_ + 0.06299 (R^2^ = 0.9651) with a LOD of 4.0 µM (Fig. 3G). The CuAuPt/Cu-TCPP(Fe)-based colorimetric system exhibited good performance in glucose detection compared with the other reported nanozyme-based colorimetric detection methods. CuAuPt/Cu-TCPP(Fe) colorimetric system has low detection limit and wide linear range. The CuAuPt/Cu-TCPP(Fe) colorimetric system has a much shorter detection time than other colorimetric methods. It is worth noting that the CuAuPt/Cu-TCPP(Fe) colorimetric system in this study simultaneously reported the linear range, detection limit and detection time of H_2_O_2_ and glucose, which was not achieved by other platforms (Table S5). To assess the selectivity of this developed method, lactose, lactic acid, uric acid, sucrose, and fructose were chosen as interfering substances. As displayed in Fig. 3H and Fig. S18, the effects of these interferences were negligible compared with glucose, suggesting the excellent selectivity of this method in glucose quantification.

To further explore if the CuAuPt/Cu-TCPP(Fe) could be applied to detect H_2_O_2_ and glucose in a complex matrix, the standard recovery experiments were applied in human serum spiked with different concentrations of H_2_O_2_ or glucose. According to the serum standard curves (Fig. S19), the average recoveries of H_2_O_2_ and glucose in serum ranged from 95.08 to 100.87% and 91.61–95.07%, with with the relative standard deviations (RSDs) within 3.77–15.66% and 0.99–8.35%, respectively (Table S6). These results showed that CuAuPt/Cu-TCPP(Fe)-based colorimetric system had appreciable accuracy and reliability, and could be applied to the detection of H_2_O_2_ and glucose.

### Application of CuAuPt/Cu-TCPP(Fe) nanozyme in portable detection

**Fig. 4:**
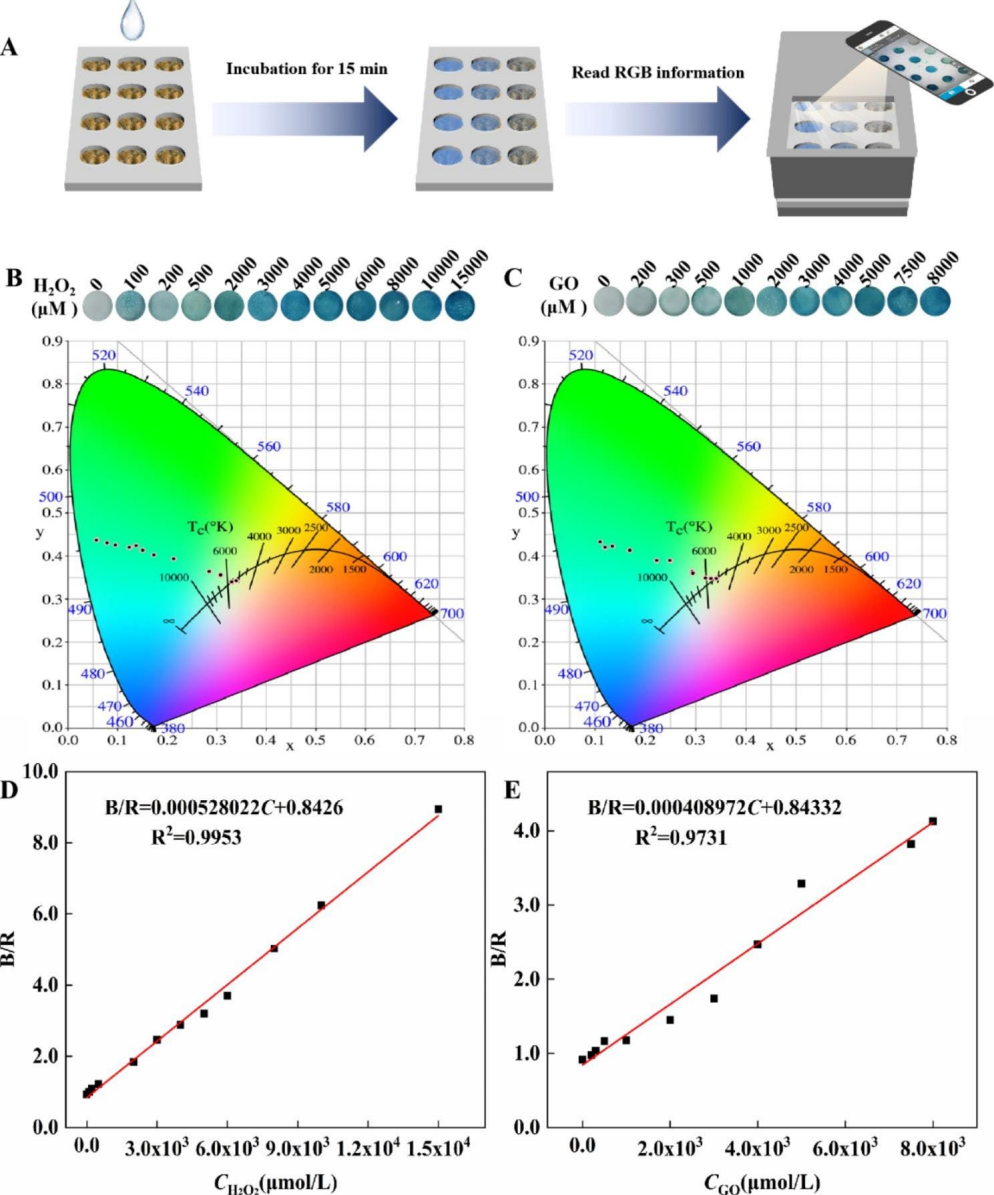
**(A)** Working principle of the visual POCT device. Photos and chromaticity spatial image of the CuAuPt/Cu-TCPP(Fe)-based test strips for the detection of **(B)** H_2_O_2_ and **(C)** glucose in serum. The linear curve of B/R for different concentrations of **(D)** H_2_O_2_ and **(E)** glucose in serum

With the increasing demands of personalized health monitoring, it is of great importance to achieve accurate and portable detection. To explore the potential application of CuAuPt/Cu-TCPP(Fe) nanozyme in POCT, the CuAuPt/Cu-TCPP(Fe) nanozyme and TMB were integrated with a filter paper to fabricate the user-friendly test strips, and then assembled with the sample box and smartphone to fabricate a visual POCT device for H_2_O_2_ and glucose detection (Fig. 4A). The full and internal view of the visual POCT device were presented in Fig. S20, in which the color changes, correlated with target concentration, can be captured and output as RGB information. As shown in Fig. 4B-C and Fig. S21, the test strips exhibited obvious color changes from colorless to blue along with an increase in the concentrations of H_2_O_2_ and Glucose. By converting the above color signals into the B/R ratio, a good linear correlation between B/R values and H_2_O_2_ concentrations was B/R = 0.000528022*C*_*H2O2*_ + 0.8426 (R^2^ = 0.9953) in the range of 0–15 mM (Fig. 4D). Similarly, a favorable linear relationship between B/R values and concentrations of glucose ranging from 0 mM to 8 mM was obtained with the linear equation of B/R = 0.000408972*C*_GO_ + 0.84332, R^2^ = 0.9731) (Fig. 4E). These results confirmed that the CuAuPt/Cu-TCPP(Fe) had great potential in POCT application. Apart from that, this visual POCT device can be applied for high-throughput analysis (measurement of multiple samples at once) without the assistance of any other expensive or complicated analytical equipments.

### Clinical serum glucose samples detection

**Fig. 5:**
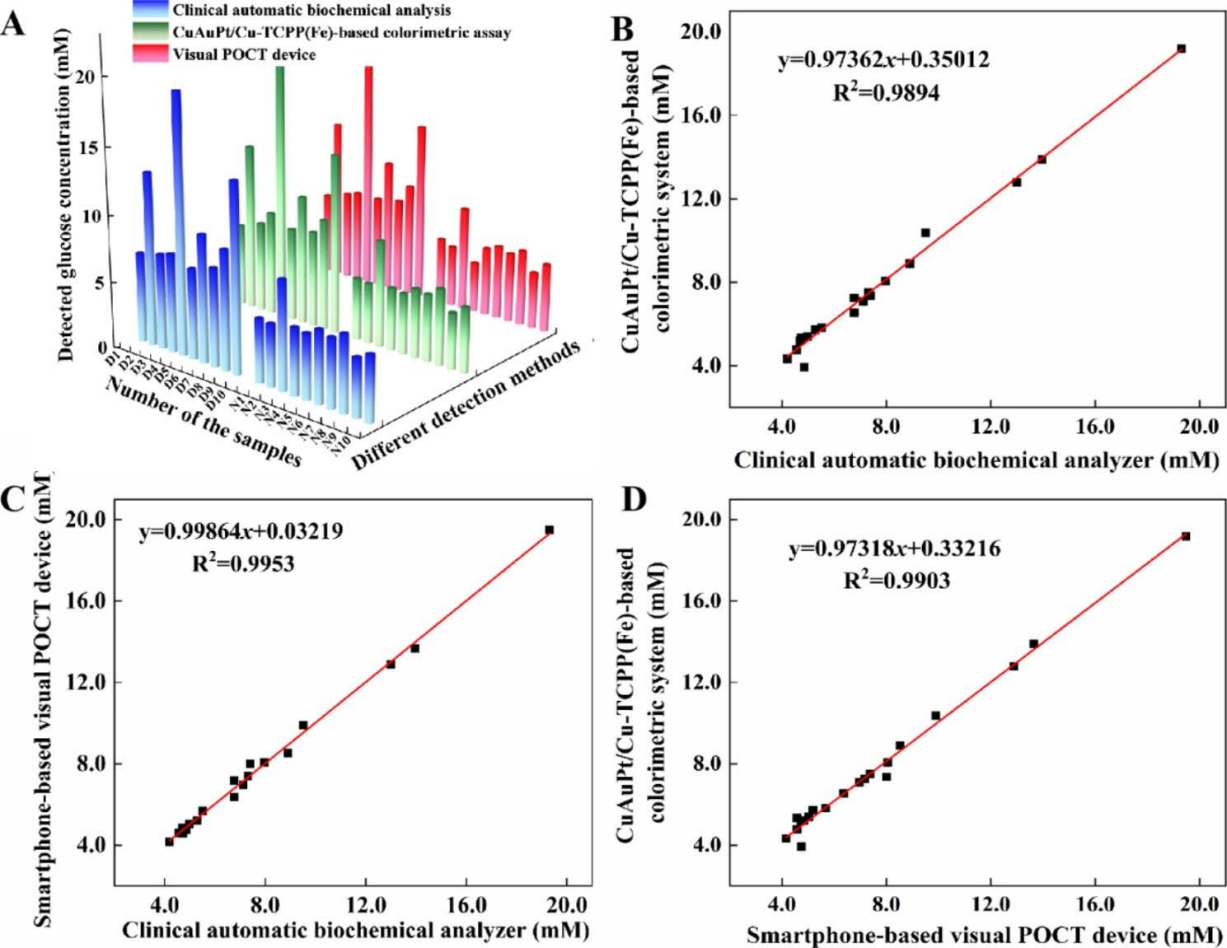
**(A)** Results of three different methods for measuring human serum glucose. Correlation curve of **(B)** the developed CuAuPt/Cu-TCPP(Fe)-based colorimetric assay with clinical automatic biochemical analysis, **(C)** the fabricated visual POCT device with clinical automatic biochemical analysis, **(D)** the developed CuAuPt/Cu-TCPP(Fe)-based colorimetric assay with the visual POCT device

The blood glucose level is a critical index for diabetes and is mainly measured in clinical laboratories. To apply our methods for clinical diagnosis, 20 clinical serum samples (10 from diabetic and 10 from healthy adults) for glucose detection were tested by the developed CuAuPt/Cu-TCPP(Fe)-based colorimetric system and the fabricated visual POCT device. The detected glucose values were compared with those obtained by clinical automatic biochemical analysis. Results showed that both in diabetic and healthy adults, there was no significant difference in glucose values measured by the above three methods (Fig. 5A and Table S7). Moreover, correlation analysis between the CuAuPt/Cu-TCPP(Fe)-based colorimetric assay and clinical automatic biochemical analysis, the visual POCT device and clinical automatic biochemical analysis, as well as the CuAuPt/Cu-TCPP(Fe)-based colorimetric assay and the visual POCT device was performed and presented in Fig. 5B-D. The results illustrated that three of them agreed well with each other with high correlation, indicating that both the developed CuAuPt/Cu-TCPP(Fe)-based colorimetric assay and the fabricated visual POCT device could serve as alternatives to clinical automatic biochemical analysis for the accurate and portable detection of serum glucose, which ultimately provide the on-site screen of diabetes and precise diagnosis of diabetic complications.

### Stability of CuAuPt/Cu-TCPP(Fe) and test strips

**Fig. 6:**
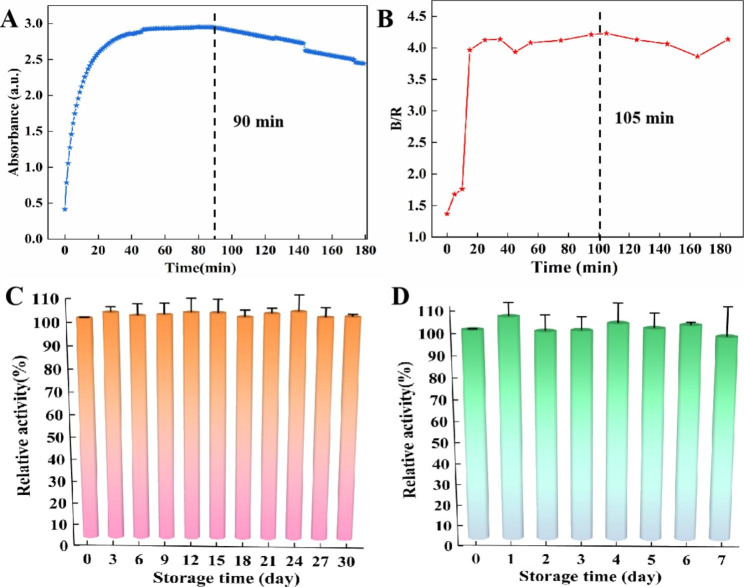
The color rendering stabilization time of **(A)** the CuAuPt/Cu-TCPP(Fe) nanozyme, and **(B)** CuAuPt/Cu-TCPP(Fe)-based test strips. The storage stability of **(C)** CuAuPt/Cu-TCPP(Fe), and **(D)** CuAuPt/Cu-TCPP(Fe)-based test strips

The color rendering stability is one of the important indicators in practical applications, therefore, the color rendering stabilization time of CuAuPt/Cu-TCPP(Fe) nanozyme and CuAuPt/Cu-TCPP(Fe)-based test strips after catalytic was studied. As presented in Fig. 6A, the A_652nm_ of CuAuPt/Cu-TCPP(Fe)-based colorimetric system in solution reached a plateau at 15 min and maintain for about 90 min, then gradually decreased. However, in the smartphone-based visual POCT system, the B/R value significantly increased within 20 min and stabilized for at least 100 min (Fig. 6B), indicating the great potential of the fabricated smartphone-based visual POCT system for practical applications.

In addition, the storage stability of CuAuPt/Cu-TCPP(Fe) and CuAuPt/Cu-TCPP(Fe)-based test strips, which played a key role in ensuring the accuracy and repeatability for the detection, were further investigated by monitoring the relative catalytic activities. As shown in Fig. 6C, the absorbance at 652 nm had almost no change, indicating that CuAuPt/Cu TCPP (Fe) nanozyme remained high-efficient enzyme catalytic activity even after 30 days of storage. At the same time, the relative catalytic activities of the test strips maintained >90% within 7 days of storage (Fig. 6D). The results implied that both the CuAuPt/Cu-TCPP(Fe) nanozyme and CuAuPt/Cu-TCPP(Fe)-based test strips possessed good storage stability. As shown in Fig. S22, CuAuPt/Cu-TCPP(Fe) maintained high relative activity at 25 ℃, 35 ℃ and 45 ℃ for 7 days. It shows that CuAuPt/Cu-TCPP(Fe) has catalytic activity at different temperatures, and can keep the properties stable.

## Conclusion

In conclusion, a multimetallic nanoparticles decorated-MOF strategy for the synthesis of the boosting peroxidase-like nanozyme was proposed. By in situ grow Cu/Au/Pt TNPs on Cu-TCPP(Fe), we successfully synthesized a novel multimetallic NP decorated-MOF (CuAuPt/Cu-TCPP(Fe)) nanozyme with significantly enhanced peroxidase-like activity. The DFT calculation revealed that the superior peroxidase-like activity of CuAuPt/Cu-TCPP(Fe) nanozyme resulted from decreased potential barriers for *OH generation in the catalytic process. Inspired by the high-efficient catalytic decomposition of H_2_O_2_ into •OH, the CuAuPt/Cu-TCPP(Fe) nanozyme achieved sensitive and selective detection of H_2_O_2_ and glucose. The practical application of the CuAuPt/Cu-TCPP(Fe)-based POCT device was successfully employed for a portable test of 20 clinical serum glucose samples, which results agreed well with clinical automatic biochemical analysis. Collectively, our work confirmed that the multimetallic NP decorated-MOF strategy was an efficient way to enhance the peroxidase-like activity of MOF-based nanozymes, and also, demonstrated the practical potential of our developed CuAuPt/Cu-TCPP(Fe) nanozyme in POCT application, which can potentially meet the requirements of personalized health monitoring and portable diagnostics.

## Electronic supplementary material

Below is the link to the electronic supplementary material.

Supplementary Material 1

Supplementary Material 2

## Data Availability

The data that supports the findings of this study are available within the article [and its supplementary material].
