# Supplementary material for: Multimetallic nanoparticles decorated metal-organic framework for boosting peroxidase-like catalytic activity and its application in point-of-care testing
Source: J Nanobiotechnology. 2023 Jun 9;21:185. doi: 10.1186/s12951-023-01946-8 (PMC10251598; doi:10.1186/s12951-023-01946-8)
Supplement: Supplementary file 1 — Supplementary Material 1 [file 12951_2023_1946_MOESM1_ESM.doc]

**Supporting Information**

**Multimetallic nanoparticles decorated metal-organic framework for boosting peroxidase-like catalytic activity and its application in point-of-care testing**

Pian Wu^a,b,1^, Fangjie Gong^a,b,1^, Yong Xia^c^, Lehuan Xia^d^, Ping Ding^a,b*^

^a^ *Xiang Ya School of Public Health, Central South University, Changsha, Hunan, 410078, China*

^b^ *Hunan Provincial Key Laboratory of Clinical Epidemiology, Changsha, Hunan, 410078, China*

^c^ *Affiliated Hospital of Xiangnan University, Chenzhou, Hunan, 423000, China*

^d^ *Chenzhou Third People's Hospital, Chenzhou, Hunan, 423000, China*

^*^Corresponding author.

E-mail address: pingshui@csu.edu.cn (P, Ding)

^1^ Pian Wu and Fangjie Gong contributed equally to this work.

**Supporting information included:**

1. Experimental Section

2. Supporting Figures

3. Supporting Tables

4. References

1. **Experimental Section**
   1. Materials and reagents

Fe(III) meso-tetra(4-carboxyphenyl)porphine chloride (TCPP(Fe)) was purchased from Macklin Biochemical Co., Ltd. (Shanghai, China). Cu(NO_3_)_2_·3H_2_O, CuSO_4_·5H_2_O, trifluoroacetic acid, polyvinylpyrrolidone, trisodium citrate, and 3,3',5,5'-tetramethylbenzidine (TMB) were purchased from Sinopharm Chemical Reagent Co., Ltd (Beijing, China). K_2_PtCl_4_ and HAuCl_4_·3H_2_O were purchased from Sigma-Aldrich Co., Ltd. (MO, USA). Glucose oxidase (GOx), glucose, hydrogen peroxide (H_2_O_2_, 30%), NaBH_4_, and terephthalic (TA) were obtained from Aladdin Biochemical Technology Co., Ltd (Shanghai, China). Whatman No. 1 chromatography paper was provided by Waterman international Ltd. (UK). Xinxing medium speed filter paper was obtained from Hangzhou Special paper Co., Ltd (Hangzhou, China). Deionized water was obtained through the Woter water purifier system (Sichuan, China). Clinical serum samples were collected from Chenzhou Third People's Hospital (Hunan, China).

- 1. Apparatus

The UV-Vis spectrophotometer (T9, Persee Co., Ltd., China) was used to record the UV-Vis absorption spectra. The scanning electron microscope (SEM, TESCAN MIRA LMS, TESCAN, Czech Republic), transmission electron microscopy (TEM, Titan G260-300, FEI, USA), atomic Force Microscope (AFM, Bruker Dimension Icon, Bruker, Germany), diffractometer (XRD, D8 Advance, Bruker, Germany), thermal gravimetric analyzer (TG, TGA 5500, TA Instruments, Inc., USA), X-ray photoelectron spectroscopy apparatus (XPS, EscaLab Xi+, Thermo Fisher, USA), and Fourier transform infrared spectrometer (FTIR, Nicolet is50, Thermo Fisher, USA) were all carried out to characterize the synthesized materials. The electron paramagnetic resonance (EPR, Bruker EMXplus-6/1, Bruker, Germany) was used to record the free radicals in during catalytic process.

- 1. Synthesis of CuAuPt/Cu-TCPP(Fe) nanozyme

Firstly, the Cu-TCPP(Fe) nanosheets were prepared according to the previously reported method and stored at ethanol[1]. The CuAuPt/Cu-TCPP(Fe) nanozyme was synthesized by situ growing the CuAuPt TNPs onto Cu-TCPP(Fe) nanosheets. In brief, 1 mL Cu-TCPP(Fe) nanosheets (1 mg/mL) was dispersed in 10 mL H_2_O, followed by the addition of 36 μL CuSO_4_·5H_2_O (0.1 M) and 75 μL trisodium citrate (0.1 M) under continuous stirring for 10 min. Then, 1.5 mL KBH_4_ (25 mM) was droply added under stirring. Subsequently, 75 μL HAuCl_4_ (0.1 M) and 75 μL K_2_PtCl_4_ (0.1 M) were addded in and keep stirring for another 20 min. The black products were collected and washed with water two times. Finally, the obtained CuAuPt/Cu-TCPP(Fe) nanozyme was stored at water.

- 1. Evaluation of peroxidase-like activity of CuAuPt/Cu-TCPP(Fe) nanozyme

The TMB was used as catalytic substrate to assess the peroxidase-like activity of CuAuPt/Cu-TCPP(Fe) nanozyme in the presence of H_2_O_2_. Tipycally, 25 μL CuAuPt/Cu-TCPP(Fe) (57 μg/L) was added into 145 μL (Na-Ac) buffer (10 mM, pH=5.5), followed by adding 20 μL TMB (6 mM) and 10 μL H_2_O_2_ (a certain concentration), then the mixture was incubated at room temperature for 15 min and the obsorbance of oxTMB was recorded at 652 nm.

For kinetic assay of CuAuPt/Cu-TCPP(Fe) nanozyme, the experiments were carried out in Na-Ac buffer (1 mM, pH=5.5) containing 7.13 μg/L CuAuPt/Cu-TCPP(Fe), 0.6 mM TMB and a series of concentrations of H_2_O_2_ in the rang of 0.25 to 2 mM, or in Na-Ac buffer (1 mM, pH=5.5) containing 7.13 μg/L CuAuPt/Cu-TCPP(Fe), 0.6 mM H_2_O_2_ and a series of concentrations of TMB in the rang of 0.08 to 1.5 mM. All experiments were repeated three times.

- 1. Hydroxyl radicals (•OH) detection

The terephthalic acid (TA) was used as a fluorescent probe to indicate the production of •OH. Five experimental groups, including TA, CuAuPt/Cu-TCPP(Fe), TA+H_2_O_2_, CuAuPt/Cu-TCPP(Fe)+H_2_O_2_, and CuAuPt/Cu-TCPP(Fe)+TA+H_2_O_2_ were evaluated at Na-Ac buffer (1 mM, pH=5.5). The experiments were carried under room temperature in the dark for 10 min with final final concentrations of TA, CuAuPt/Cu-TCPP(Fe), and H_2_O_2_ were 0.2 mM, 7.13 μg/L, and 2 mM, respectively. The maximum emission fluorescence peaks at 425 nm of 2-hydroxy terephthalic acid (TA-OH), the oxidation product of TA, were record.

In addition, the tert-Butanol (TBA) was used as the trapper to consume the produced •OH. Four experimental groups, including TMB+TBA, TMB+TBA+H_2_O_2_, CuAuPt/Cu-TCPP(Fe)+TMB+H_2_O_2_, and CuAuPt/Cu-TCPP(Fe)+TMB+TBA+H_2_O_2_ were evaluated at Na-Ac buffer (1 mM, pH=5.5). The experiments were carried under room temperature for 15 min with final final concentrations of TMB, TBA, CuAuPt/Cu-TCPP(Fe), and H_2_O_2_ were 0.6 mM, 1 mM, 7.13 μg/L, and 0.5 mM, respectively. The maximum absorbance peaks at 652 nm of oxTMB were record.

- 1. DFT calculation

The first-principles calculation steps were completed through structural optimization, static self-consistent field (SCF), density of state calculation, and charge calculation. All calculation steps are completed by VASP-6.1.01[2], combined with the PBE functional[3, 4] under the generalized gradient approximation (GGA)[5](Becke 1996) in the frame of density functional theory (DFT) with D3 dispersion correction[6], combined with the projector augmented wave (PAW)[7], and the plane wave cut off energy is 700 eV. Among them, K point adopts the density of 7*7*7 for bulk materials and 7*7*1 for surface.

- 1. Detection of H_2_O_2_ and glucose in solution

For the detection of H_2_O_2_, different concentrations of H_2_O_2_ solution were added in Na-Ac buffer (1 mM, pH=5.5) containing CuAuPt/Cu-TCPP(Fe) (7.13 μg/L) and TMB (0.6 mM), respectively. The absorption peak at 652 nm was measured after 15 min incubation at room temperature.

For the detection of glucose, firstly, 10 μL glucose oxidase (8 mg/mL) was incubated with 290 μL of glucose solution at 37 ℃ for 15 min. Then, 20 μL glucose incubation solution was mixed with 25 μL of CuAuPt/Cu-TCPP(Fe) (57 μg/L), 20 μL of the TMB (6 mM), and 135 μL of the Na-Ac buffer solution (10 mM, pH=5.5). The mixture was incubated for another 15 min and was used for the absorption spectrum measurement.

- 1. Fabrication of visual POCT device

The visual POCT device was fabricated through two steps, including the preparation of CuAuPt/Cu-TCPP(Fe)-based test strips and the optical detection device. The CuAuPt/Cu-TCPP(Fe)-based test strips were prepared as follows: the Whatman No. 1 filter paper was cut into a 1 cm disc and was immersed into Cu/Au/Pt-MOFs (57 μg/L) dispersions for 30 min, followed by drying at 60 ℃. Then, 100 μL Na-Ac buffer solution (pH 5.5, 10 mM) and 50 μL chitosan solution (0.25 % wt) were dropped onto that Cu/Au/Pt-MOFs-loaded paper in sequence. Finally, 30 μL TMB (6 mM) was dropped in and the modified test strips were dried for further usage in paper-based detection. The optical detection device was made up of a 3D printed sample box, a pull-out sample plate, a light-emitting diode (LED), and smartphone. The design of this optical detection device was shown in Fig. S1. The sample box, a 116 mm (length) × 86 mm (width) × 120 mm (height) box, was obtained by 3D printing by using black resin as the printing material, which could prevent light interference from the surrounding environment. The LED lights (1.6 W) were evenly fixed on the inner side of the sample box cover to provide a stable light source for detection. The sample plate was made of white polylactic acid (PLA) material with a tightly stitched handle and had 12 sample slots (diameter=1 cm) for placing test strips, which meet the requirements of high throughput detection. The smartphone (HUAWEI P30) was installed with Color Grab App (Fig. S2) to collect the RGB (red, green, blue) values of samples. The standard curve could be easily obtained by plotting B/R ratio versus target concentrations. Thus the concentration of target in the sample can be calculated according to the standard linear equation.

- 1. Visual quantification of H_2_O_2_ and glucose by visual POCT device

For H_2_O_2_ detection, 50 μL H_2_O_2_ solution was dropped in the test strips. After 15 min reaction, the RGB information was recorded by the developed visual POCT device. For glucose detection, 290 μL glucose solution was firstly incubated with 10 μL glucose (8 mg/mL) oxidase at 37 ℃ for 15 min. Then, 50 μL glucose incubation solution was dropped into the test strips and kept at room temperature for another 15 min, the RGB information of the test strips was read by the visual POCT device.

- 1. Detection of H_2_O_2_ and glucose in real serum samples

Human serum samples were supplied by the 10 diabetic and 10 healthy from Chenzhou Third People's Hospital (Hunan, China). The serum samples were diluted 10-fold before analysis and then determined according to the above method. All the experiments were performed in accordance with the relevant laws and institutional guidelines.

1. **Supporting Figures S1-S20**

**
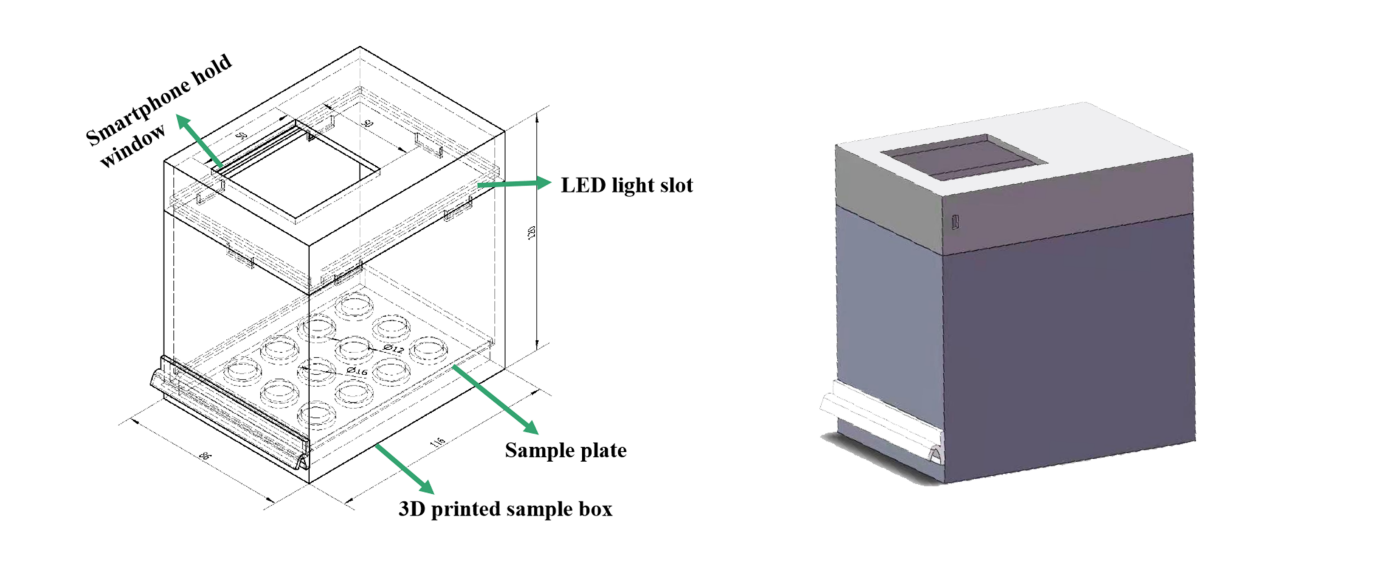
**

**Fig. S1.** The design diagram of the optical detection device.


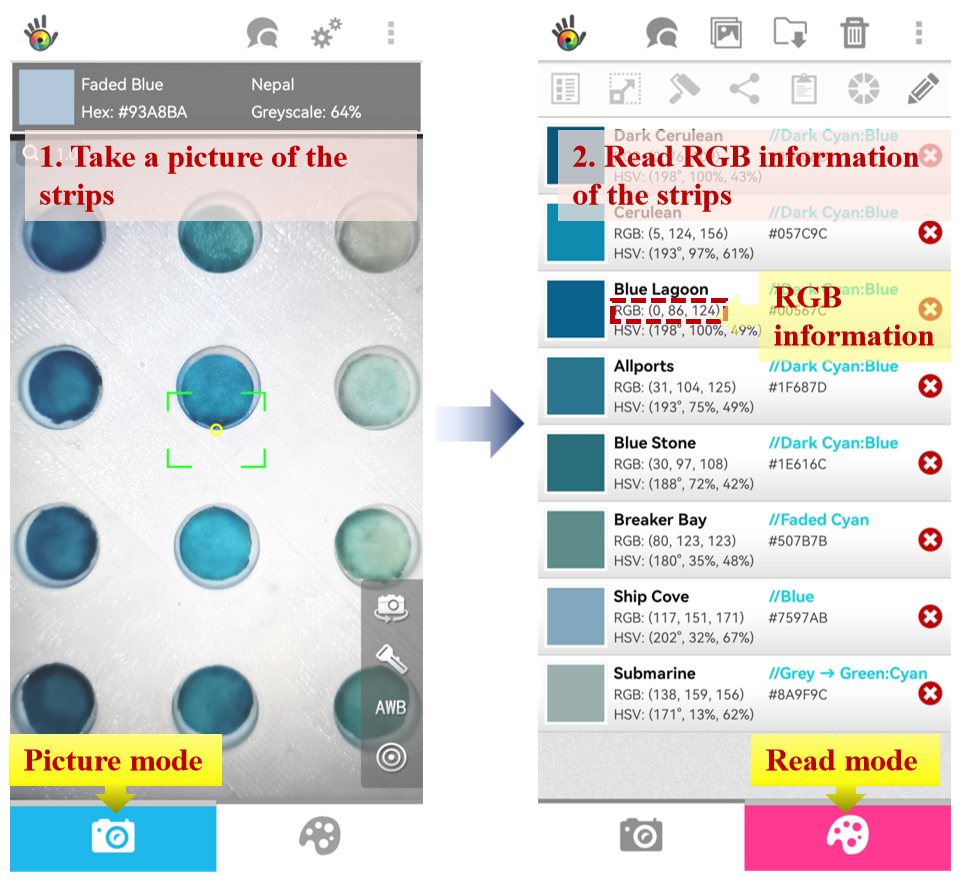


**Fig. S2.** User interface of the ColorGrab App.


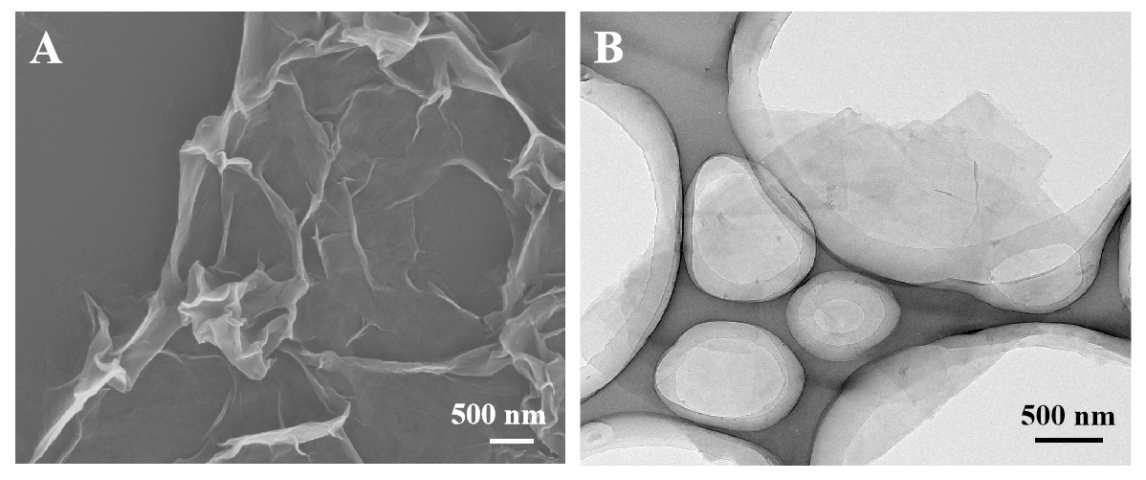


**Fig. S3.** (A) SEM and (B) TEM images of Cu-TCPP(Fe).


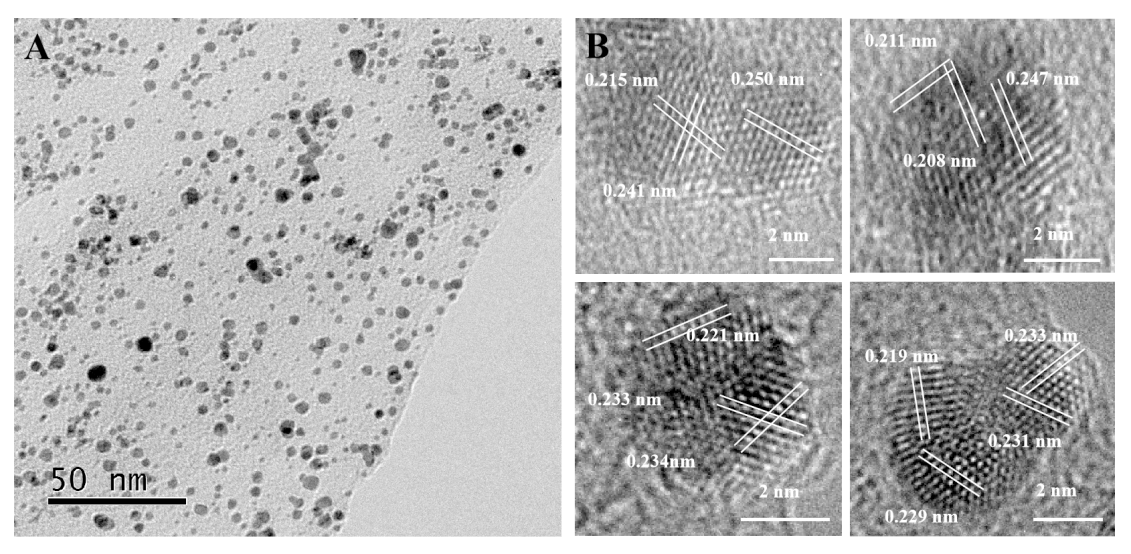


**Fig. S4.** (A) TEM and (B) HRTEM images of CuAuPt/Cu-TCPP(Fe) nanozyme.


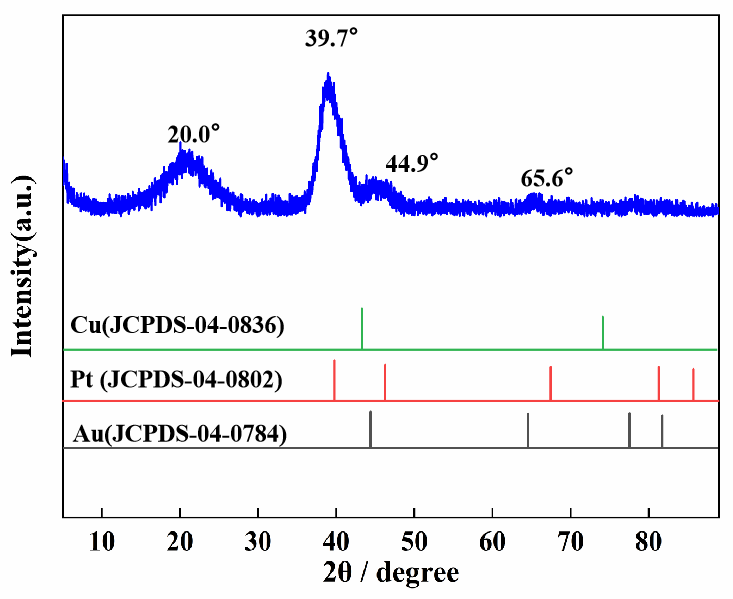


**Fig. S5.** XRD pattern of CuAuPt/Cu-TCPP(Fe) nanozyme.

**
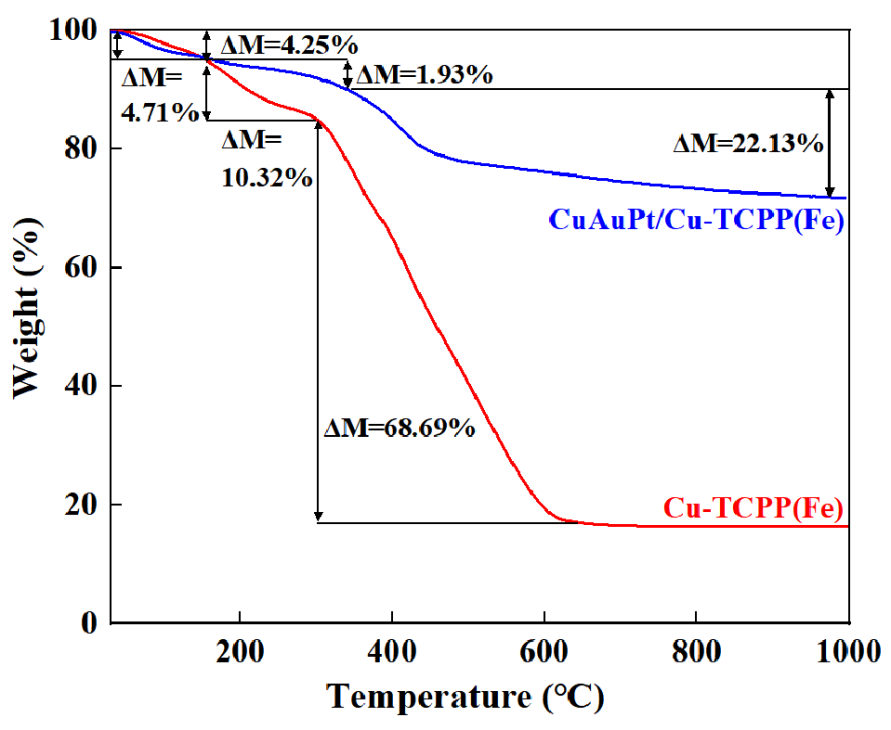
**

**Fig. S6.** TG patterns of Cu-TCPP(Fe) and CuAuPt/Cu-TCPP(Fe) nanozyme.


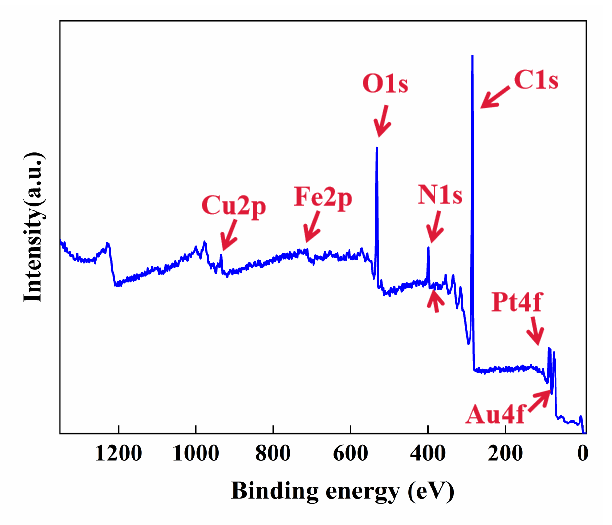


**Fig. S7.** XPS survey spectra of CuAuPt/Cu-TCPP(Fe) nanozyme.


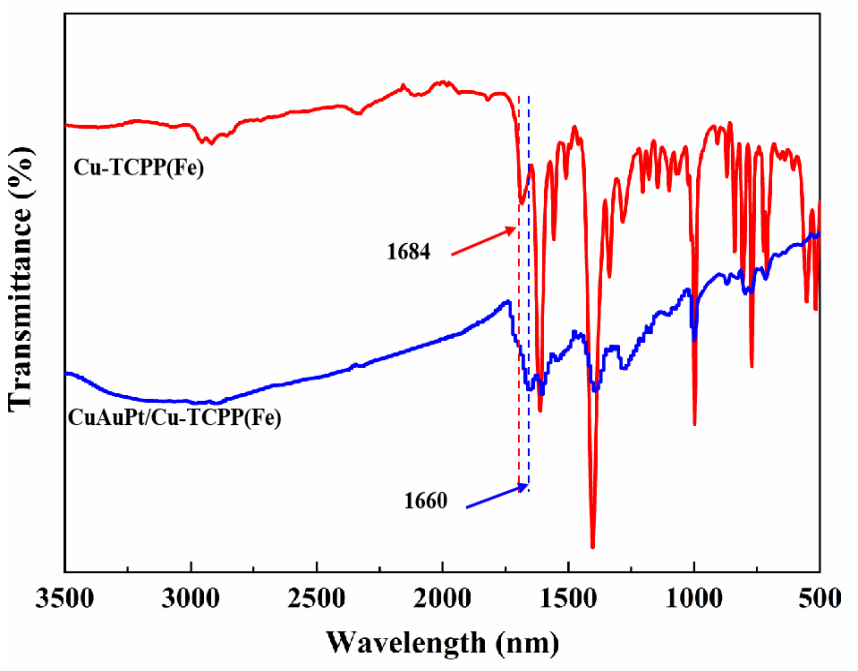


**Fig. S8.** FTIR spectra of Cu-TCPP(Fe) and CuAuPt/Cu-TCPP(Fe).


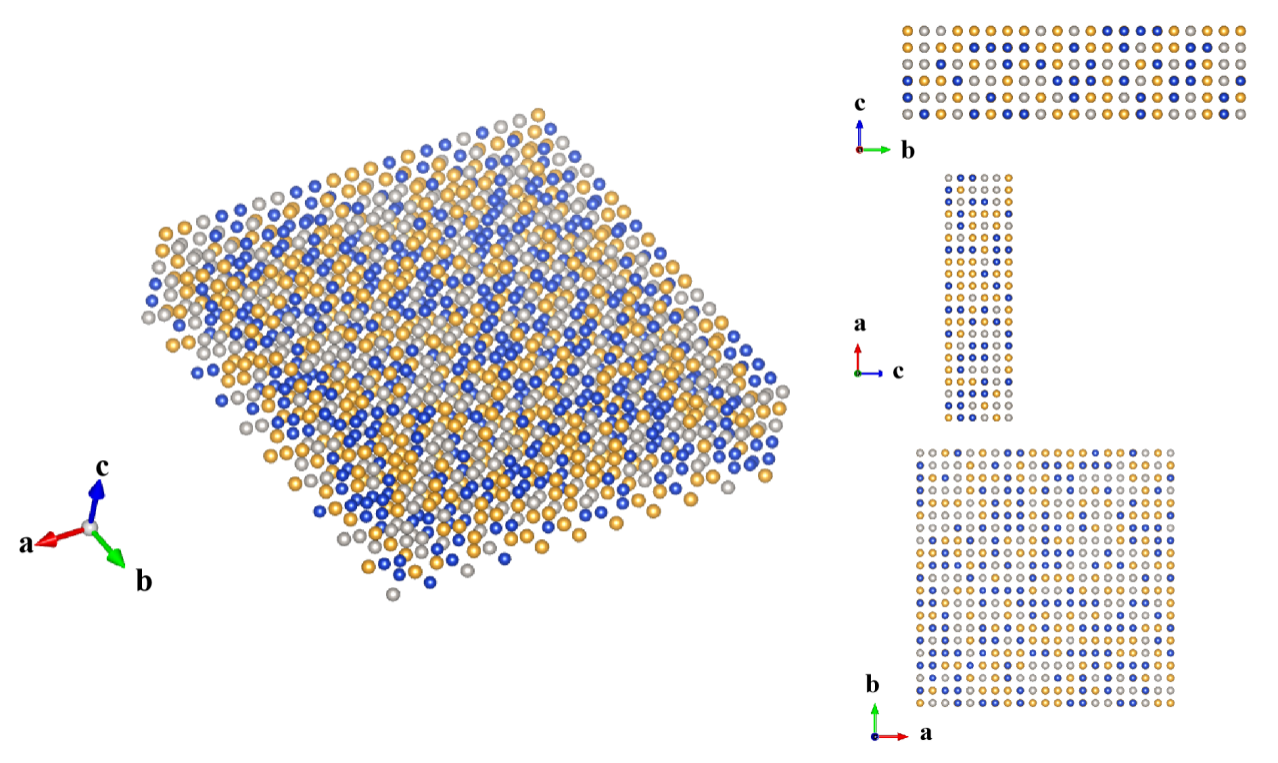


**Fig. S9.** Optimized structures of Cu/Au/Pt TNPs.

**
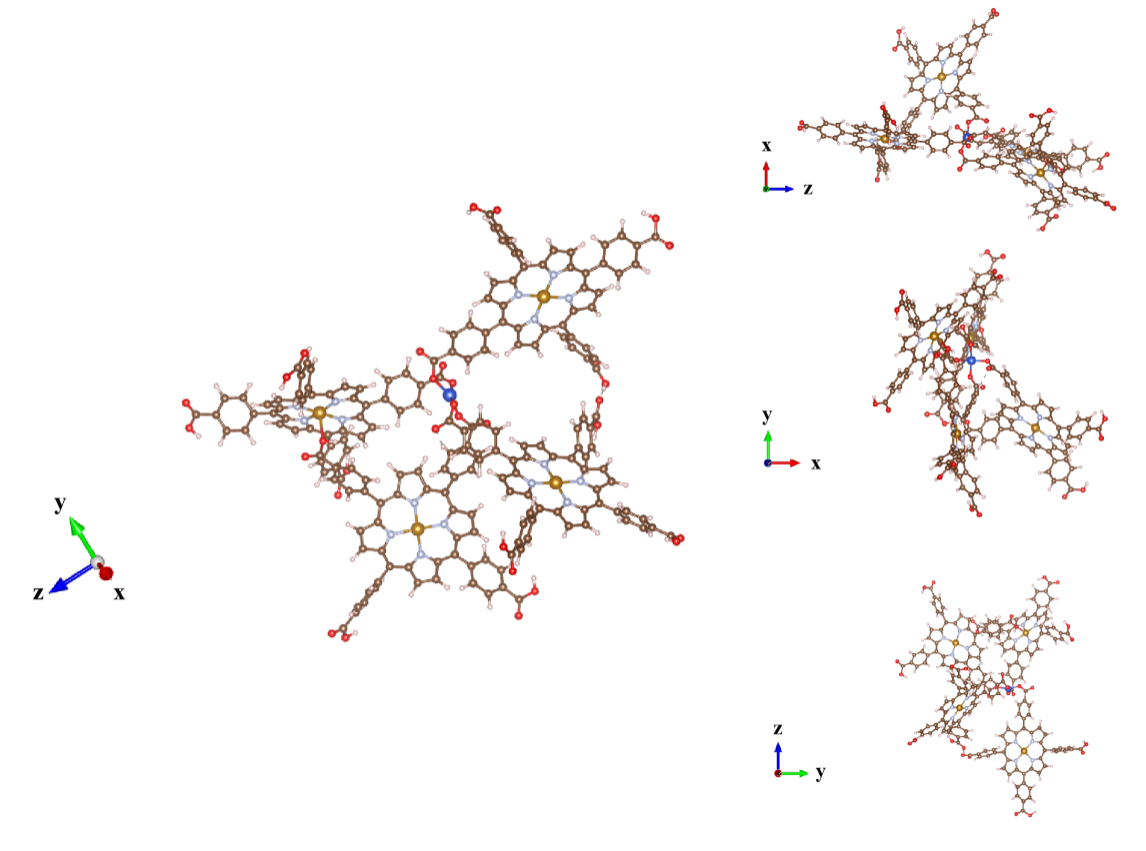
**

**Fig. S10.** Optimized structures of Cu-TCPP(Fe).


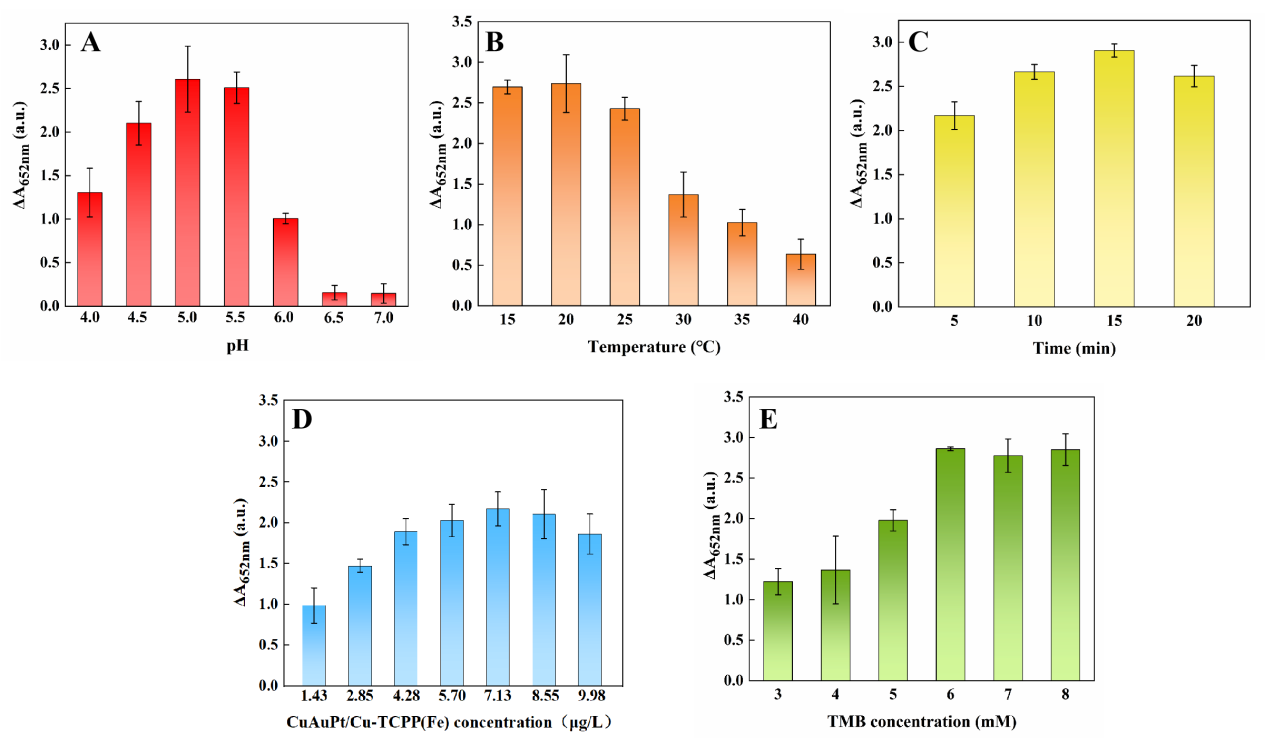


**Fig. S11.** Optimization of reaction conditions for H_2_O_2_ detection. (A) pH. (B) Temperature. (C) Reaction time. (D) The concentration of CuAuPt/Cu-TCPP(Fe), and (E) The concentration of TMB.

In order to obtain a better catalytic condition for CuAuPt/Cu-TCPP(Fe) nanozyme, the potential influence factors including pH, temperature, reaction time, the concentrations of CuAuPt/Cu-TCPP(Fe) nanozyme and TMB were optimized. The pH was firstly studied since pH had a significant effect on the peroxidase-like activity of nanozymes. From Fig. S11A, it can be seen that when the pH was less than 5, the catalytic activity increased with the increase of pH, and reached the highest at pH of 5. Notably, there was no significant difference in the catalytic activity between pH=5 and pH=5.5, but a smaller error was found in pH=5.5, so pH=5.5 was chosen as the optimal condition. Fig. S11B showed that the CuAuPt/Cu-TCPP(Fe) nanozyme maintained high catalytic activity between 15-25 ℃, suggesting that CuAuPt/Cu-TCPP(Fe) nanozyme had good catalytic activity at room temperature. In addition, the optimized reaction time was 15 min, and the concentrations of TMB and CuAuPt/Cu-TCPP(Fe) nanozyme were 0.6 mM and 7.13 μg/L, respectively (Fig. S11C-E).


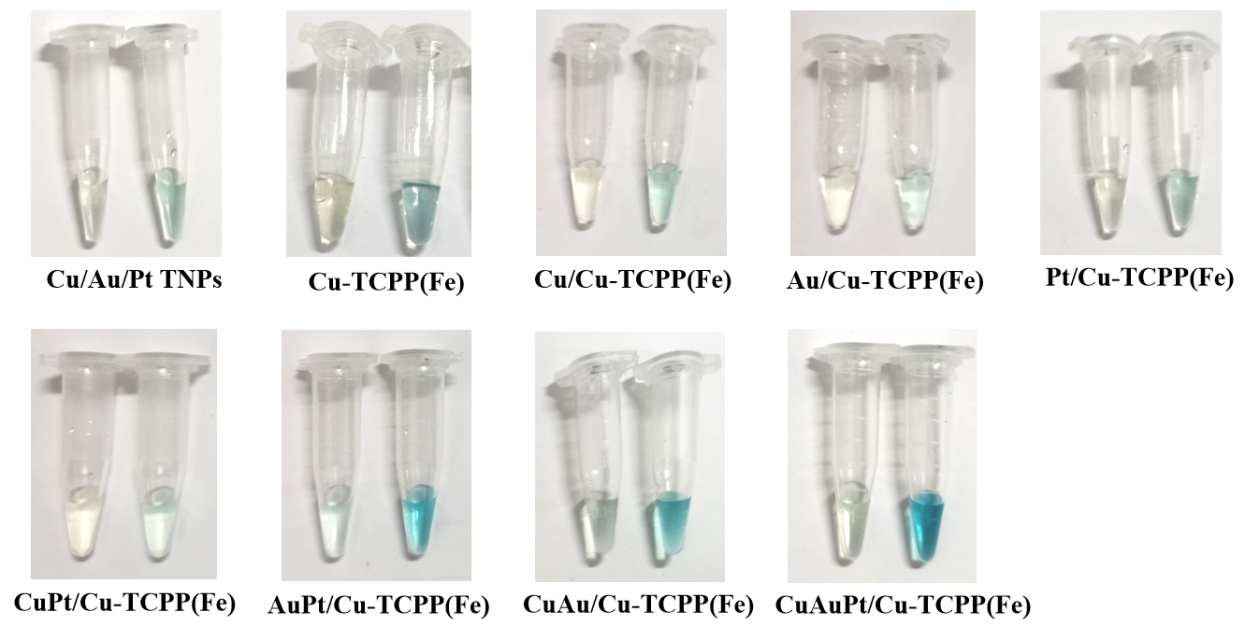


**Fig. S12.** Photos of TMB oxidation by different materials.


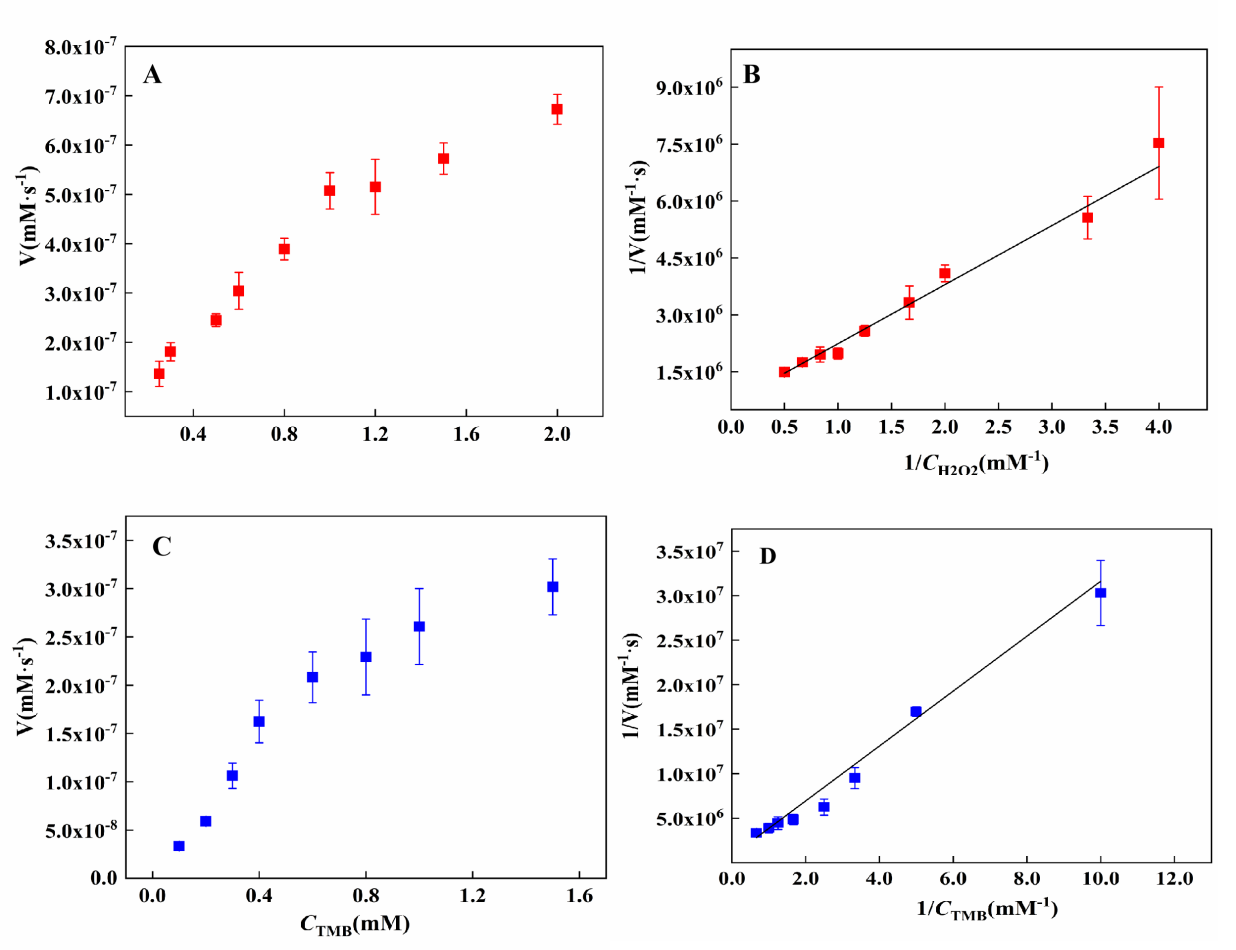


**Fig. S13.** (A) Michaelis-Menten kinetic analysis and (B) Lineweaver-Burk plotting for CuAuPt/Cu-TCPP(Fe) nanozyme with H_2_O_2_ as a substrate. (C) Michaelis-Menten kinetic analysis and (D) Lineweaver-Burk plotting for CuAuPt/Cu-TCPP(Fe) nanozyme with TMB as a substrate.


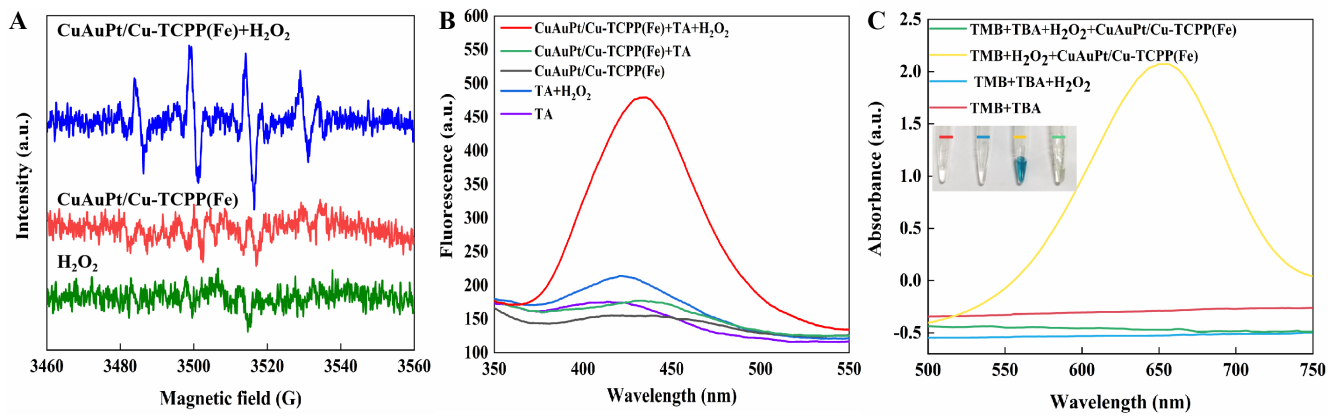


**Fig. S14.** Mechanism of CuAuPt/Cu-TCPP(Fe) as boosting peroxidase-like nanozyme. (A) EPR spectra of •OH by DMPO spin-trapping in different systems. (B) Fluorescence spectra of different groups. (C) UV-vis spectra of TMB of different groups.


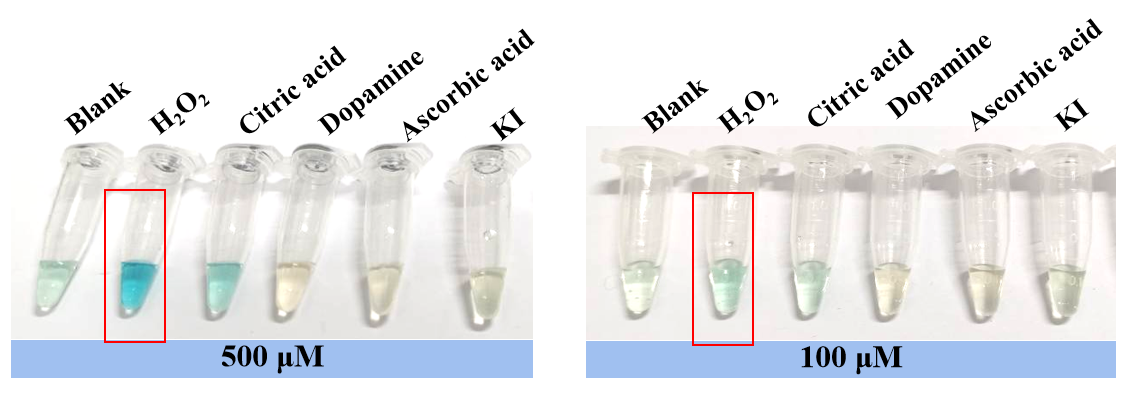


**Fig. S15.** Photos of interferences in CuAuPt/Cu-TCPP(Fe)-based colorimetric assay for the detection of H_2_O_2_.


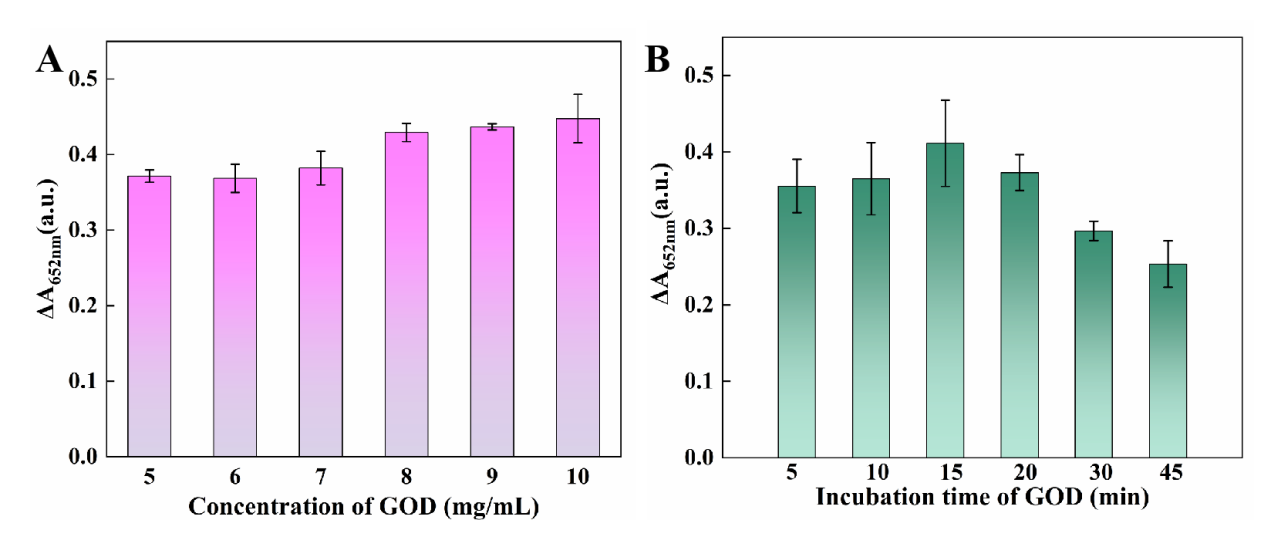


**Fig. S16.** Optimization of reaction conditions for glucose detection. (A) GOx concentration. (B) Incubation time.

**
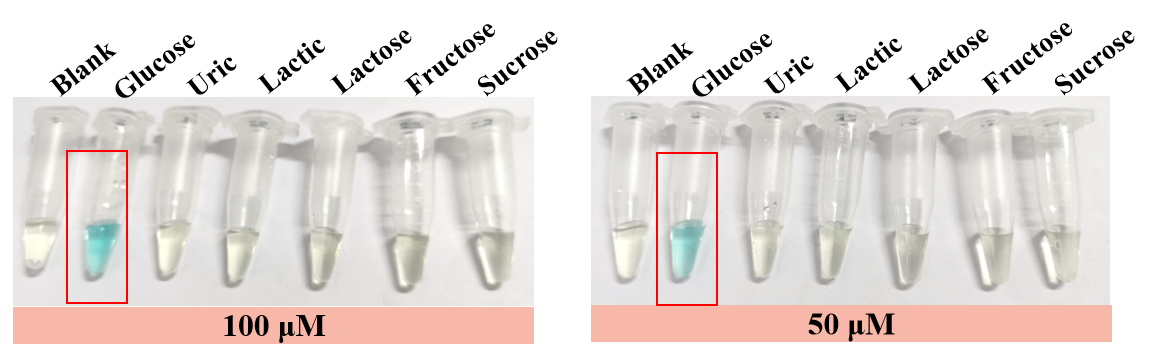
**

**Fig. S17.** Photos of interferences in CuAuPt/Cu-TCPP(Fe)-based colorimetric assay for the detection of glucose.


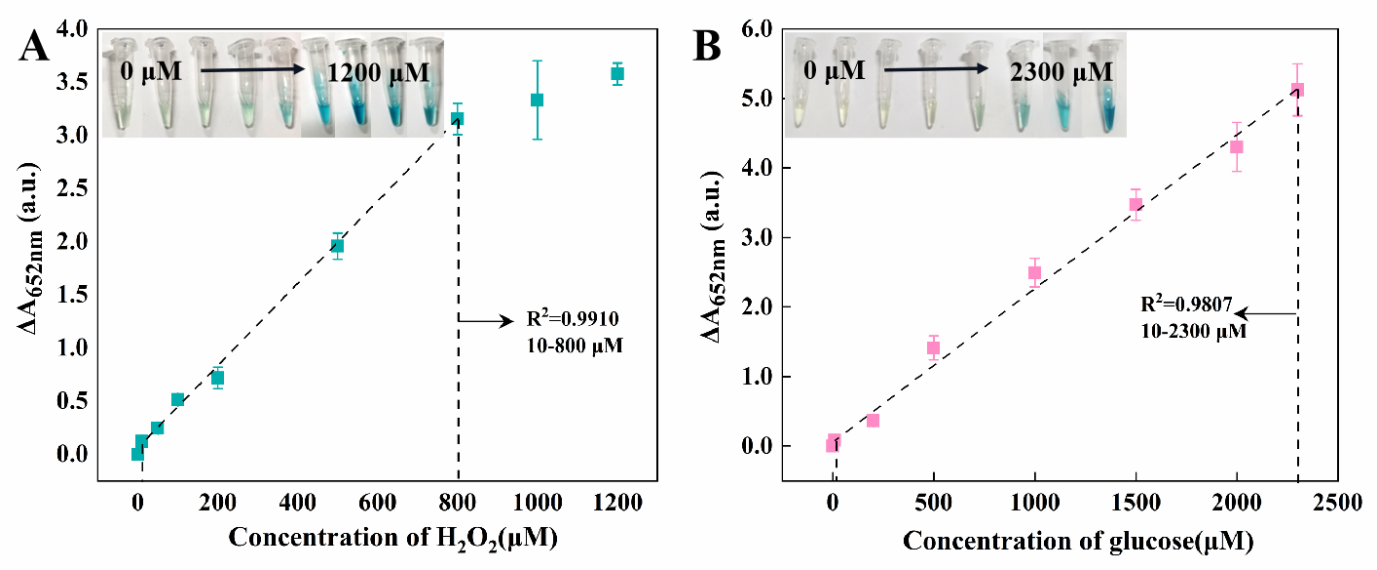


**Fig. S18.** The linear curve of Δ_652nm_ for different (A) H_2_O_2_, and (B) glucose concentrations in the serum matrix, inset: photos of TMB in presence of different H_2_O_2_ and glucose concentrations.


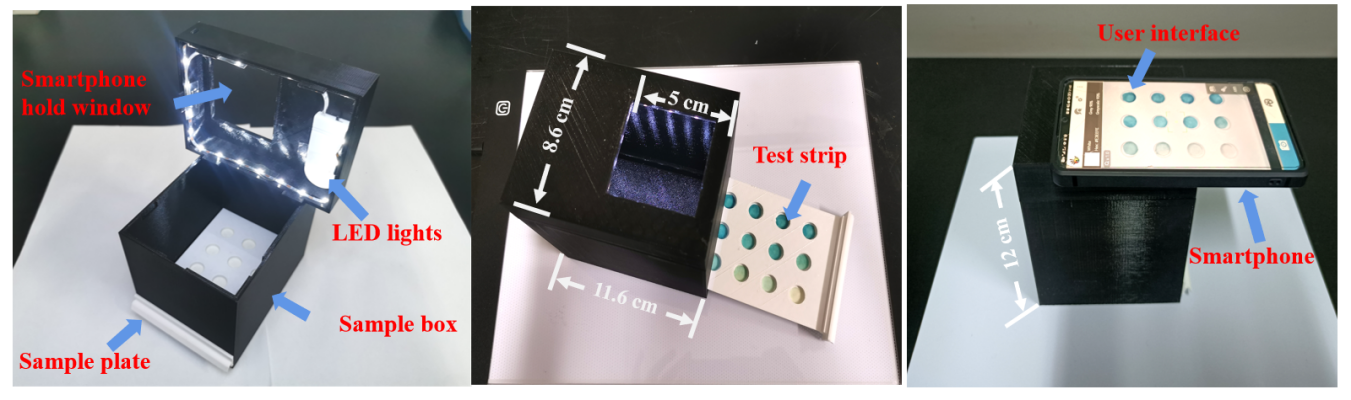


**Fig. S19.** The full view and internal view of the visual POCT device.


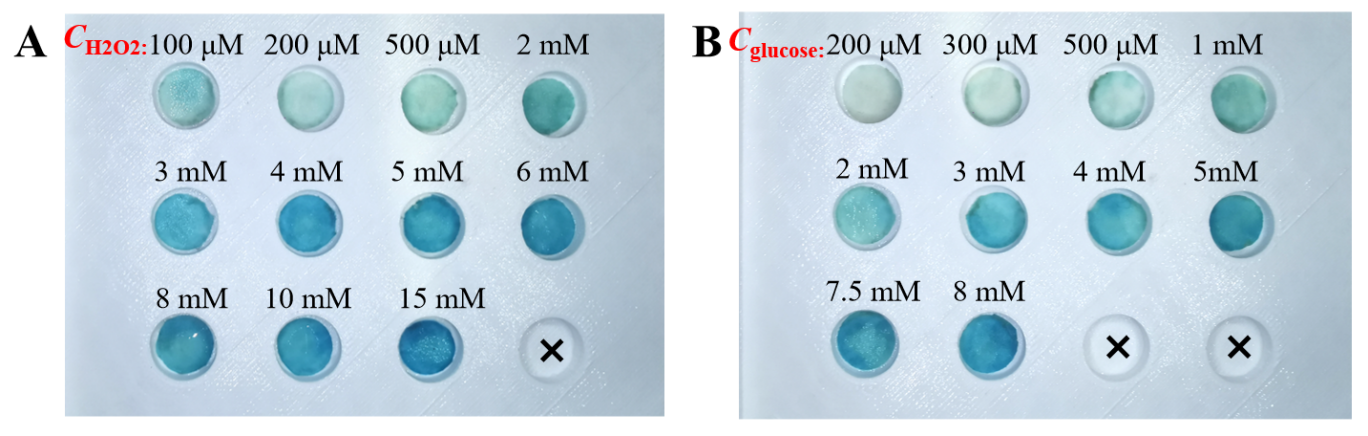


**Fig. S20.** Photos of oxTMB in presence of different (A) serum H_2_O_2_ concentrations, and (B) serum glucose in the test strips.

**Supporting Tables S1-S7**

**Table S1.** Metal elements concentration of CuAuPt/Cu-TCPP(Fe).

|  | **Fe** | **Cu** | **Au** | **Pt** |
| --- | --- | --- | --- | --- |
| **CuAuPt/Cu-TCPP(Fe)** | 0.287 mg/L | 1.356 mg/L | 4.140 mg/L | 5.069 mg/L |

**Table S2.** XPS data for CuAuPt/Cu-TCPP(Fe).

|  | **Fe** | **Cu** | **Au** | **Pt** |
| --- | --- | --- | --- | --- |
| **Metallic state** | - | Cu^0^/Cu^+^ (43.3 %) | Au^0^ (92.62 %) | Pt^0^ (79.54 %) |
| **Oxidized state** | Fe^3+^ (100 %) | Cu^+^ (16.06 %)  Cu^2+^ (40.65 %) | Au^+^ (7.38 %) | Pt^+^ (20.46 %) |

**Table S3.** Comparison of Michaelis kinetics for different nanozymes.

|  | **Substrate** | **K_m_ (mM)** | **V_max_ (M s^-1^)** | **Refs.** |
| --- | --- | --- | --- | --- |
| CoO-OMC | H_2_O_2_ | 2.66×10^-14^ | 3.29×10^-3^ | [8] |
|  | TMB | - | - |  |
| FeCu@2-DG | H_2_O_2_ | 17.70 | 2.98×10^-5^ | [9] |
|  | TMB | 0.81 | - |  |
| PL-CsPbBr_3_ NCs | H_2_O_2_ | 2.85 | 5.85×10^-8^ | [10] |
|  | TMB | 1.42 | 6,21×10^-8^ |  |
| CuO | H_2_O_2_ | 400 | 0.3×10^-8^ | [11] |
|  | TMB | 25 | 2.87×10^-8^ |  |
| Cu-TA-350 | H_2_O_2_ | 1.27×10^3^ | 1.40×10^-5^ | [12] |
|  | TMB | - | - |  |
| HRP | H_2_O_2_ | 3.70 | 8.71×10^-8^ | [13] |
|  | TMB | 0.43 | 10.00×10^-8^ |  |
| CuAuPt/Cu-TCPP(Fe) | H_2_O_2_ | 2.28 | 1.46×10^-6^ | This work |
|  | TMB | 0.0040 | 1.31×10^-6^ |  |

**Table S4.** Reaction free energy of intermediate species on Cu-TCPP(Fe) and CuAuPt/Cu-TCPP(Fe).

| **Free energy**  **Catalyts** | **H_2_O_2_** | **﹡OOH** | **﹡OH** | **H_2_O** |
| --- | --- | --- | --- | --- |
| **Cu-TCPP(Fe)** | 0 | -1.323 | -1.241 | -1.472 |
| **CuAuPt/Cu-TCPP(Fe)** | 0 | -1.429 | -1.362 | -1.683 |

**Table S5.** Comparison of the developed system with reported methods for the detection of H_2_O_2_ and glucose.

| **Probe** | **Method** | **H_2_O_2_** | | | **glucose** | | | **Refs.** |
| --- | --- | --- | --- | --- | --- | --- | --- | --- |
|  |  | **Linear range (μM)** | **LOD (μM)** | **Detection time (min)** | **Linear range (μM)** | **LOD (μM)** | **Detection time (min)** |  |
| ZnO TP/MXene | Electrochemical | - | - | - | 50-700 | 17 | - | [14] |
| indium tin oxides (ITO) | Electrochromic | 0-500 | - | 0.5 | 0-200 | 123 | 0.5 | [15] |
| GNPs@linoleic acid | fluorescence spectroscopy | - | - | - | 0-16650 | 2800 | - | [16] |
| Fe_0.96_O_0.88_(OH)_1.12_ | Colorimetric | 1-6000 | 60 | - | 1-6000 | 30 | 60 | [17] |
| Ti^3+^- ATNTs | Photoelectrochemical | - | - | - | 100-6000 | - | - | [18] |
| rGO/GOx | Electrochemical |  |  |  | 1000-25000 | 320 | - | [19] |
| four-layer CeO_2_ woodpile | Electrochemical |  |  |  | 20-2500 | 10 | 1440 | [20] |
| Fe_3_O_4_-Gr | SPR measurement | - | - | - | 100-10000 | 199.5 | 3 | [21] |
| glass/ITO/ZnO-PDA/GOx | Photoelectrochemical | - | - | - | 15-120 | 6.2 | - | [22] |
| ZnO/ LIG | Colorimetric | 800-32700 | 110 | 190 | - | - | - | [23] |
| Zn-Co-OH/3DNF | Electrochemical | - | - | - | 10-3535 | 8.33 | - | [24] |
| CoO-OMC | Colorimetric | 10-700; 1000-5000 | - | 30 | 100-5000 | 68 | 60 | [8] |
| La-MCM-41 | Colorimetric | - | - | - | 50-1000 | 37.5 | 70 | [25] |
| CuAuPt/Cu-TCPP(Fe) | Colorimetric | 10-800 | 9.32 | 15 | 10-500 | 3.95 | 30 | This work |

**Table S6. Detection of H_2_O_2_ and glucose in complex matrix using CuAuPt/Cu-TCPP(Fe)-based colorimetric system (n = 3).**

| **Substance** | **Matrix** | **Spiked (μM)** | **Detected (μM)** | **Recovery (%)** | **RSD (%)** |
| --- | --- | --- | --- | --- | --- |
| H_2_O_2_ | Serum | 100 | 100.87 | 100.87 | 3.77 |
|  |  | 500 | 490.95 | 98.19 | 15.66 |
|  |  | 800 | 760.66 | 95.08 | 1.00 |
| Glucose | Serum | 200 | 190.13 | 95.07 | 8.35 |
|  |  | 1000 | 916.14 | 91.61 | 0.99 |
|  |  | 2000 | 1866.52 | 93.33 | 4.92 |

**Table S7. Determination of glucose in clinical serum samples.**

| **Sample number** | **Detected glucose values (mM)** | | |
| --- | --- | --- | --- |
|  | Clinical automatic biochemical analysis | CuAuPt/Cu-TCPP(Fe)-based colorimetric system | The visual POCT device |
| D1 | 6.77 | 6.54 | 6.374 |
| D2 | 13.01 | 12.79 | 12.88 |
| D3 | 7.12 | 7.08 | 6.96 |
| D4 | 7.4 | 7.35 | 8.00 |
| D5 | 19.31 | 19.19 | 19.49 |
| D6 | 6.77 | 7.26 | 7.18 |
| D7 | 9.51 | 10.37 | 9.90 |
| D8 | 7.31 | 7.49 | 7.39 |
| D9 | 8.9 | 8.89 | 8.53 |
| D10 | 13.97 | 13.89 | 13.66 |
| N1 | 4.70 | 5.18 | 4.73 |
| N2 | 4.56 | 4.76 | 4.59 |
| N3 | 7.97 | 8.06 | 8.06 |
| N4 | 4.86 | 3.93 | 4.75 |
| N5 | 4.73 | 5.33 | 4.58 |
| N6 | 5.29 | 5.73 | 5.19 |
| N7 | 4.98 | 5.39 | 5.04 |
| N8 | 5.52 | 5.82 | 5.69 |
| N9 | 4.20 | 4.33 | 4.17 |
| N10 | 4.70 | 5.21 | 4.86 |

**References**

1.Wu P, Ye X, Wang D, Gong F, Wei X, Xiang S, Zhang J, Kai T, Ding P: A novel CRISPR/Cas14a system integrated with 2D porphyrin metal-organic framework for microcystin-LR determination through a homogeneous competitive reaction. *Journal of Hazardous Materials* 2022; 424:127690.

2.Hafner J: Ab-initio simulations of materials using VASP: Density-functional theory and beyond. *Journal of Computational Chemistry* 2008; 29:2044-2078.

3.Kresse G, Joubert D: From ultrasoft pseudopotentials to the projector augmented-wave method. *Physical Review B* 1999; 59:1758-1775.

4.Perdew JP, Burke K, Ernzerhof M: Generalized Gradient Approximation Made Simple. *Physical Review Letters* 1996; 77:3865-3868.

5.Becke AD: Density‐functional thermochemistry. IV. A new dynamical correlation functional and implications for exact‐exchange mixing. *The Journal of Chemical Physics* 1996; 104:1040-1046.

6.Grimme S, Antony J, Ehrlich S, Krieg H: A consistent and accurate ab initio parametrization of density functional dispersion correction (DFT-D) for the 94 elements H-Pu. *The Journal of Chemical Physics* 2010; 132:154104.

7.Blöchl PE: Projector augmented-wave method. *Physical Review B* 1994; 50:17953-17979.

8.Guo Y, Yan L, Zhang R, Ren H, Liu A: CoO-supported ordered mesoporous carbon nanocomposite based nanozyme with peroxidase-like activity for colorimetric detection of glucose. *Process Biochemistry* 2019; 81:92-98.

9.Singh R, Zeng Q, Cheng S, Kumar S: Selective Colorimetric Detection of Cancer Cells Based on Iron/Copper Nanocatalyst Peroxidase Activity. *IEEE Sensors Journal* 2022; 22:10492-10499.

10.Li M, Wang Y, Hu H, Feng Y, Zhu S, Li C, Feng N: A dual-readout sandwich immunoassay based on biocatalytic perovskite nanocrystals for detection of prostate specific antigen. *Biosens Bioelectron* 2022; 203:113979.

11.Nagvenkar AP, Gedanken A: Cu0.89Zn0.11O, A New Peroxidase-Mimicking Nanozyme with High Sensitivity for Glucose and Antioxidant Detection. *ACS Appl Mater Interfaces* 2016; 8:22301-22308.

12.Cheng D, Qin J, Feng Y, Wei J: Synthesis of Mesoporous CuO Hollow Sphere Nanozyme for Paper-Based Hydrogen Peroxide Sensor. *Biosensors (Basel)* 2021; 11.

13.Wu P, Ding P, Ye X, Li L, He X, Wang K: One-pot synthesized Cu/Au/Pt trimetallic nanoparticles as a novel enzyme mimic for biosensing applications. *RSC Advances* 2019; 9:14982-14989.

14.Myndrul V, Coy E, Babayevska N, Zahorodna V, Balitskyi V, Baginskiy I, Gogotsi O, Bechelany M, Giardi MT, Iatsunskyi I: MXene nanoflakes decorating ZnO tetrapods for enhanced performance of skin-attachable stretchable enzymatic electrochemical glucose sensor. *Biosens Bioelectron* 2022; 207:114141.

15.Yeon SY, Seo M, Kim Y, Hong H, Chung TD: Paper-based electrochromic glucose sensor with polyaniline on indium tin oxide nanoparticle layer as the optical readout. *Biosens Bioelectron* 2022; 203:114002.

16.Nasehi M, Saeedi M, Ghanavi J, Moravvej-Farshi MK: Glucose Sensing Based on the Interaction of Gold Nanoparticles@Linoleic Acid With the Glucose. *IEEE Sensors Journal* 2022; 22:7169-7176.

17.Abagana AY, Zhao M, Alshahrani MY, Rehman KU, Andleeb S, Wang J, Bukhari SM: Hydrogen iron oxide from an Acinetobacter strain exhibiting intrinsic peroxidase-like activity and its catalytic mechanism and applications. *Biomass Conversion and Biorefinery* 2022:1-10.

18.Chahrour KM, Ooi PC, Eid AM, Nazeer AA, Madkour M, Dee CF, Wee MFMR, Hamzah AA: Synergistic effect of bi-phased and self-doped Ti+3 on anodic TiO2 nanotubes photoelectrode for photoelectrochemical sensing. *Journal of Alloys and Compounds* 2022; 900.

19.Soganci K, Bingol H, Zor E: Simply patterned reduced graphene oxide as an effective biosensor platform for glucose determination. *Journal of Electroanalytical Chemistry* 2021; 880.

20.Zhou Y, Uzun SD, Watkins NJ, Li S, Li W, Briseno AL, Carter KR, Watkins JJ: Three-Dimensional CeO2 Woodpile Nanostructures To Enhance Performance of Enzymatic Glucose Biosensors. *ACS Appl Mater Interfaces* 2019; 11:1821-1828.

21.Rahayu TOC, Septiani NLW, Gumilar G, Adhika DR, Suyatman, Yuliarto B: Modification of Gold Substrate With Fe3O4-Graphene Nanocomposite to Increase Resolution of Surface Plasmon Resonance (SPR) Glucose Sensor. *IEEE Sensors Journal* 2021; 21:19959-19966.

22.Fedorenko V, Damberga D, Grundsteins K, Ramanavicius A, Ramanavicius S, Coy E, Iatsunskyi I, Viter R: Application of Polydopamine Functionalized Zinc Oxide for Glucose Biosensor Design. *Polymers (Basel)* 2021; 13:2918.

23.Zanoni J, Moura JP, Santos NF, Carvalho AF, Fernandes AJS, Monteiro T, Costa FM, Pereira SO, Rodrigues J: Dual Transduction of H2O2 Detection Using ZnO/Laser-Induced Graphene Composites. *Chemosensors* 2021; 9.

24.Tran TH, Thi MLN, Son NT, Bui QB, Ai-Le PH, Nhac-Vu HT: Novel nanoneedle structures of zinc-doped cobalt hydroxide as a self-supported sensor for sensitive glucose detection. *Materials Research Bulletin* 2019; 120.

25.Jabariyan S, Zanjanchi MA, Arvand M, Sohrabnezhad S: Colorimetric detection of glucose using lanthanum-incorporated MCM-41. *Spectrochim Acta A Mol Biomol Spectrosc* 2018; 203:294-300.
